# Supplementary material for: Loss of vegetation functions during the Paleocene–Eocene Thermal Maximum
Source: Nat Commun. 2025 Nov 27;16:11369. doi: 10.1038/s41467-025-66390-8 (PMC12728184; doi:10.1038/s41467-025-66390-8)
Supplement: Supplementary file 1 — Supplementary Information [file 41467_2025_66390_MOESM1_ESM.pdf]

Loss of vegetation functions during the Paleocene–Eocene Thermal Maximum

Julian Rogger[1,2*], Vera A. Korasidis[3], Gabriel J. Bowen[4], Christine A. Shields[5], Taras V. Gerya[1], Loïc Pellissier[2,6]

[1]Department of Earth and Planetary Sciences, ETH Zurich, Zurich, Switzerland.
[2]Department of Environmental Systems Science, ETH Zurich, Zurich, Switzerland.
[3]School of Geography, Earth and Atmospheric Sciences, University of Melbourne, Melbourne, Australia.
[4]Department of Geology and Geophysics, University of Utah, Salt Lake City, Utah, USA.
[5]NSF National Center for Atmospheric Research, Boulder, Colorado, USA.
[6]Swiss Federal Institute for Forest, Snow and Landscape Research, Birmensdorf, Switzerland.

*Corresponding author(s). E-mail(s): jul.rogger@gmail.com;

Supplementary Figures

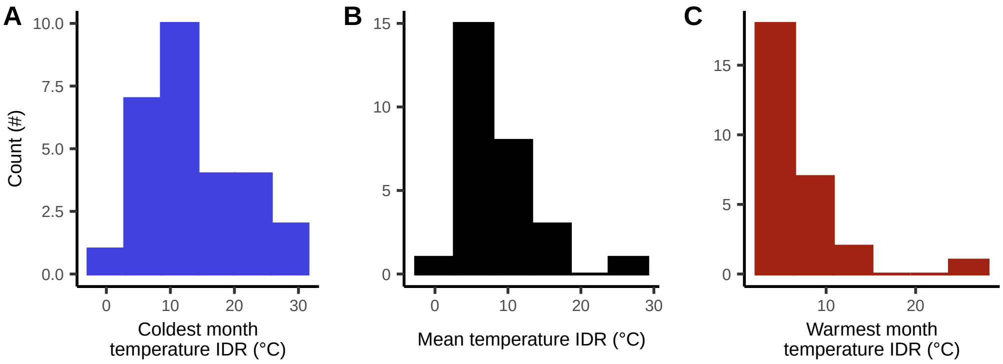

Supplementary Figure 1 Temperature variation in present-day Koeppen-Geiger zones. Koeppen-Geiger zones classify 30 different geographic regions on the globe with common climate and vegetation characteristics [1]. The variation in the coldest month, warmest month and mean temperature observed within present-day vegetation zones is used as an approximation of the temperature variation tolerable by the modelled plants in the vegetation model TREED (Trait Ecology and Evolution model over Deep time), assuming that it approximates the climatic range suitable for plants with similar trait combinations. **A.** Interdecile range (10th to 90th percentile) of the average temperature of the coldest month within Koeppen-Geiger zones. **B.** Interdecile range of the mean annual temperature within Koeppen-Geiger Zones. **C.** Interdecile range of the average temperature of the warmest month within Koeppen-Geiger zones. Overall, most Koeppen-Geiger zones exhibit an interdecile range of < 10°C in the coldest month, warmest month and mean annual temperature, which is used as the baseline temperature variation tolerable by the modelled plants. Koeppen-Geiger zones (0.5° resolution) from [1]; monthly average temperatures (1981–2010; 0.5° resolution) from CHELSA (Climatologies at High resolution for the Earth's Land Surface Areas) [2]. Figure produced using R [3] and the ggplot2 package [4].

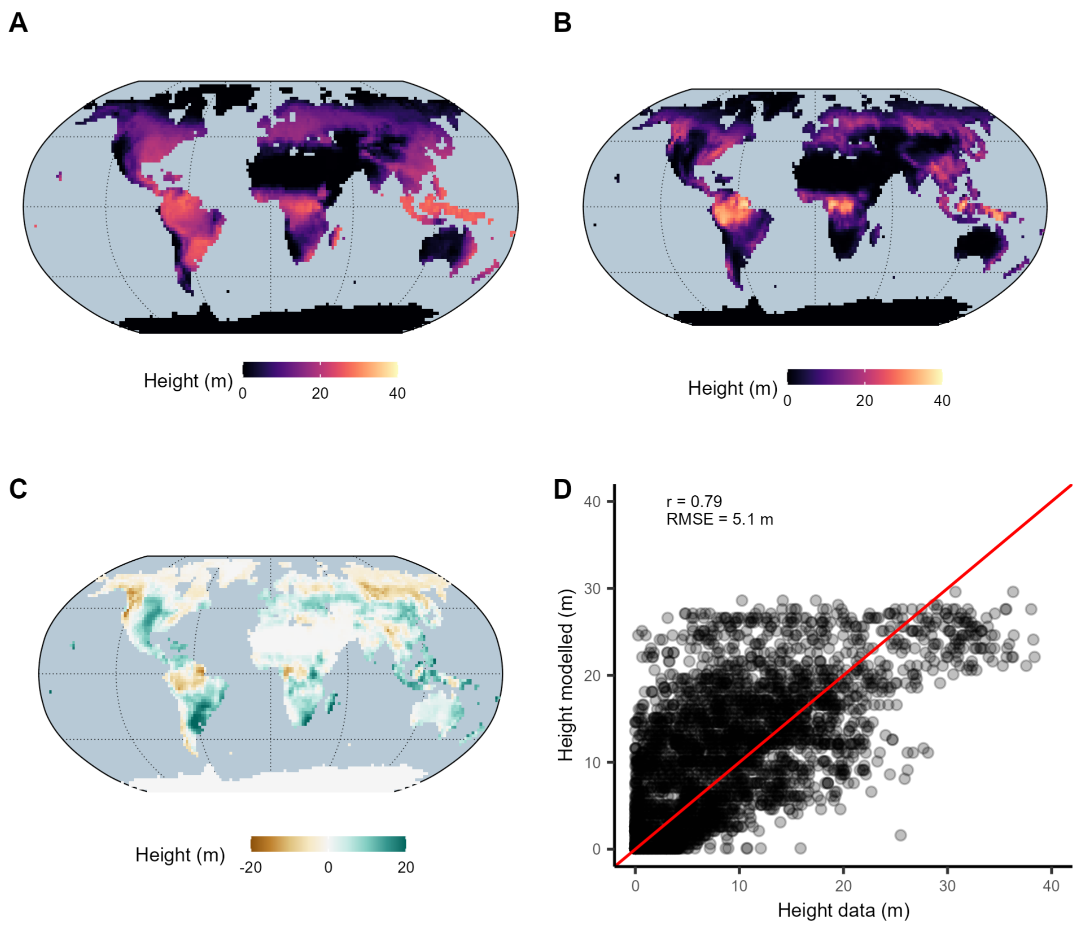

Supplementary Figure 2 Height validation of the Trait Ecology and Evolution vegetation model over Deep time (TREED) considering present-day boundary conditions. A. Modelled height distribution, **B.** satellite data and machine-learning-derived estimate of vegetation height for the year 2020 [5]. **C.** Difference between model and data (model - data). **D.** Scatterplot of modelled and observed vegetation height, the red line indicating the 1:1 line. *RMSE* stands for root mean square error and r represent the pearson correlation coefficient between modelled and observed values. Climate data for the modelling from [2] and [6]. Figure produced using R [3] and the ggplot2 package [4].

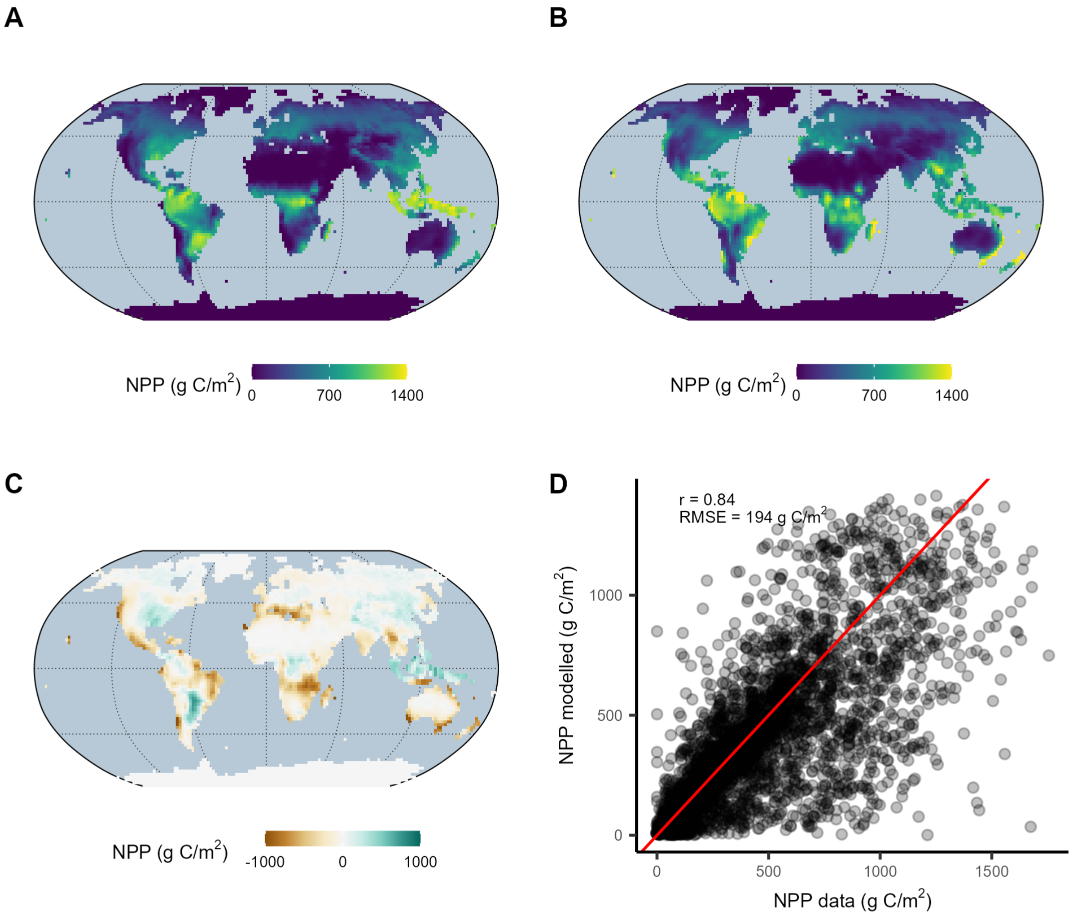

Supplementary Figure 3 Net primary productivity (NPP) validation of the Trait Ecology and Evolution vegetation model over Deep time (TREED) considering present-day boundary conditions. A. Modelled NPP distribution, **B.** NPP estimate derived from MODIS (Moderate Resolution Imaging Spectroradiometer), considering annual average fluxes for the years 2001–2010 [7]. **C.** Difference between model and data (model - data). **D.** Scatterplot of modelled and observed NPP with the red line indicating the 1:1 line. *RMSE* stands for root mean square error and r represent the pearson correlation coefficient between modelled and observed values. Climate data for the modelling from [2] and [6]. Figure produced using R [3] and the ggplot2 package [4].

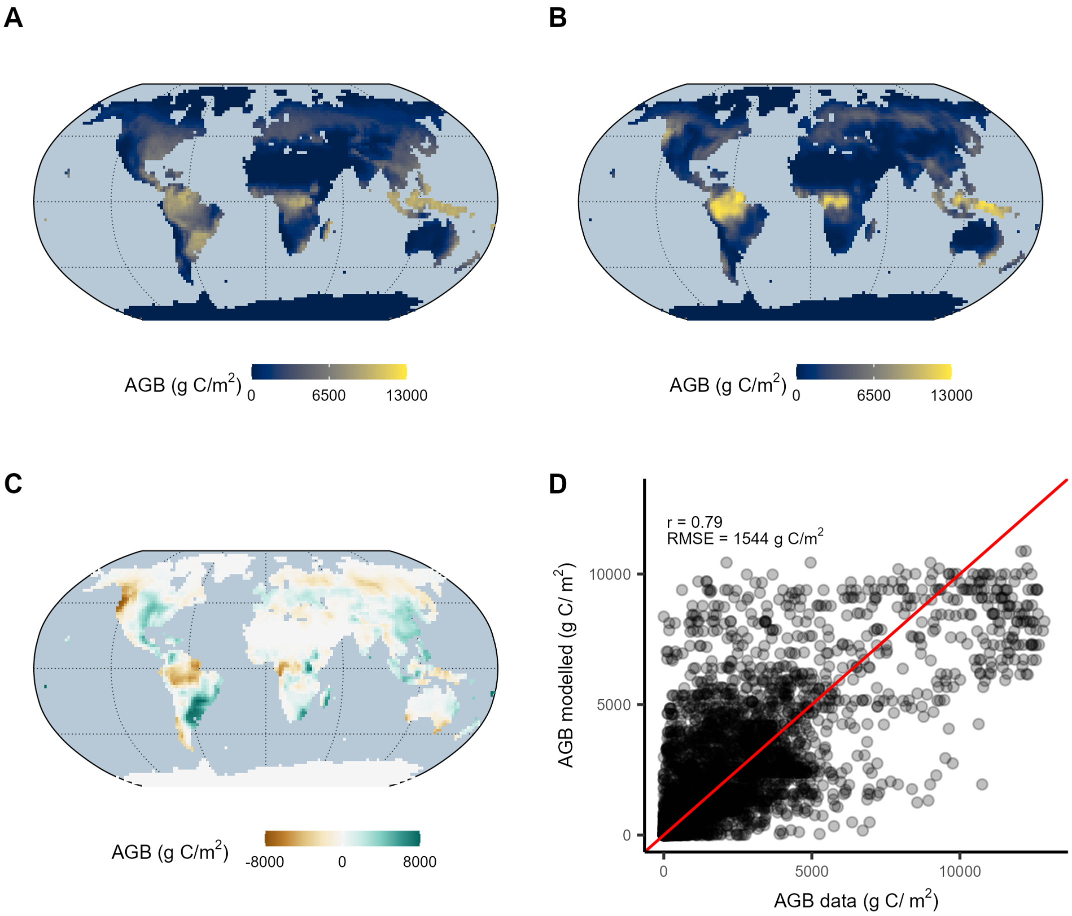

Supplementary Figure 4 Aboveground biomass (AGB) validation of the Trait Ecology and Evolution vegetation model over Deep time (TREED) considering present-day boundary conditions. **A.** Modelled AGB distribution, **B.** AGB distribution derived from a machine-learning-based global upscaling of field measurements [8]. **C.** Difference between model and data (model - data). **D.** Scatterplot of modelled and observed AGB with the red line indicating the 1:1 line. *RMSE* stands for root mean square error and r represent the pearson correlation coefficient between modelled and observed values. Climate data for the modelling from [2] and [6]. Figure produced using R [3] and the ggplot2 package [4].

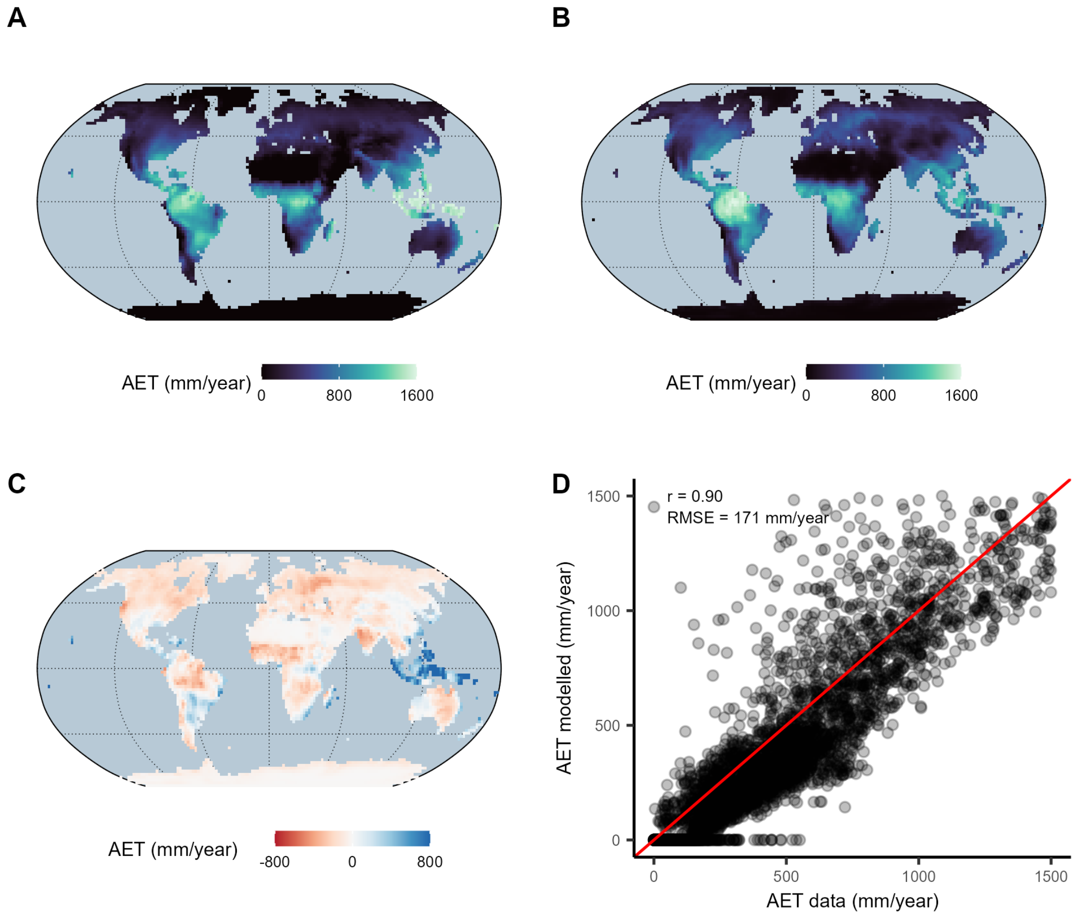

Supplementary Figure 5 Actual evapotranspiration (AET) validation of the Trait Ecology and Evolution vegetation model over Deep time (TREED) considering present-day boundary conditions. A. Modelled AET distribution, **B.** AET derived from the Global Land Evaporation Amsterdam Model for the year 2010 [9]. **C.** Difference between model and data (model - data). **D.** Scatterplot of modelled and observed AET with the red line indicating the 1:1 line. *RMSE* stands for root mean square error and r represent the pearson correlation coefficient between modelled and observed values. Climate data for the modelling from [2] and [6]. Figure produced using R [3] and the ggplot2 package [4].

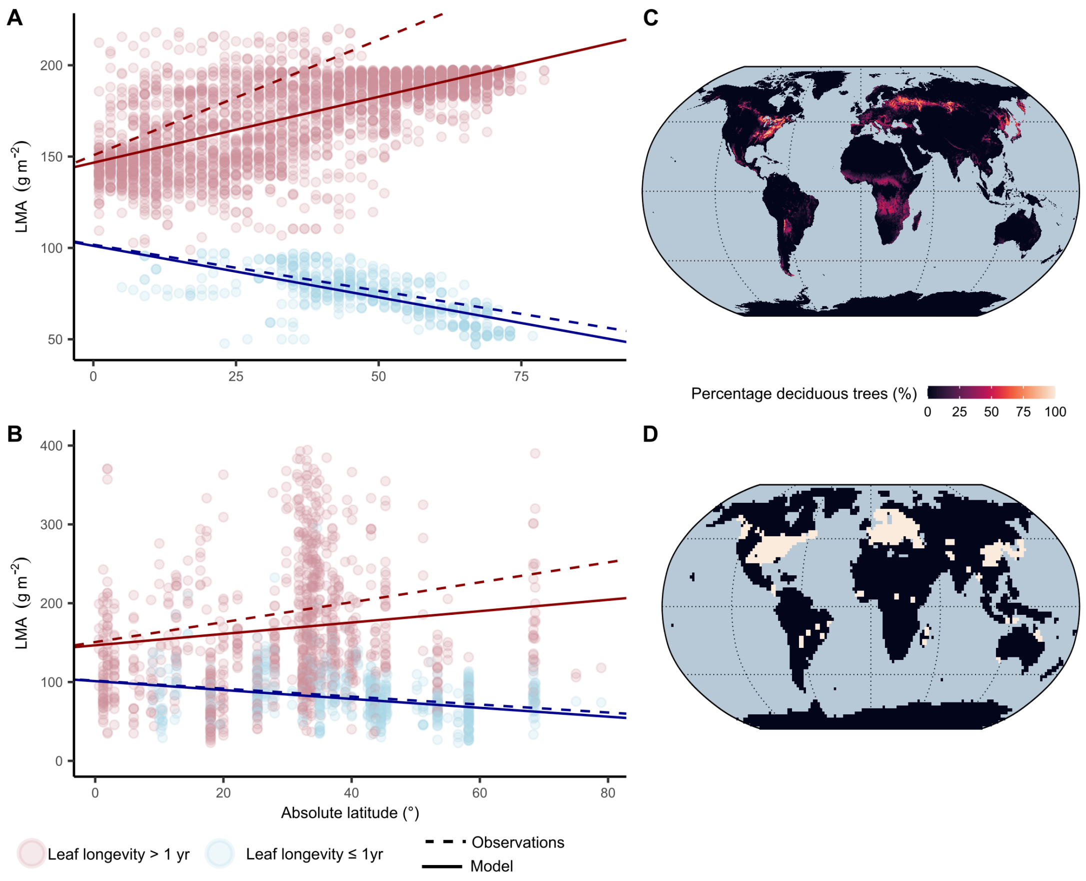

Supplementary Figure 6 Leaf mass per area (LMA) validation of the Trait Ecology and Evolution vegetation model over Deep time (TREED) considering present-day boundary conditions. A. Modelled latitudinal trends in LMA for evergreens (leaf longevity > 1 year) and deciduous plants (leaf longevity $\leq$ 1 year). The solid line indicates a linear regression between LMA and latitude as derived from the model. The dashed line indicates a linear regression between LMA and latitude as derived from observations. Observed data from the global plant trait network (Glopnet) data set [10, 11]. **B.** Observed LMA trends across latitude with the model-derived and data-derived regression. Mid-latitude LMA values above 400 g m^{-2} were removed for the visualisation, but kept in the regression. **C.** Observed contribution of deciduous trees to vegetation composition, Data from [12]. **D.** Modelled distribution of deciduous phenology. Climate data for the modelling from [2] and [6]. Figure produced using R [3] and the ggplot2 package [4].

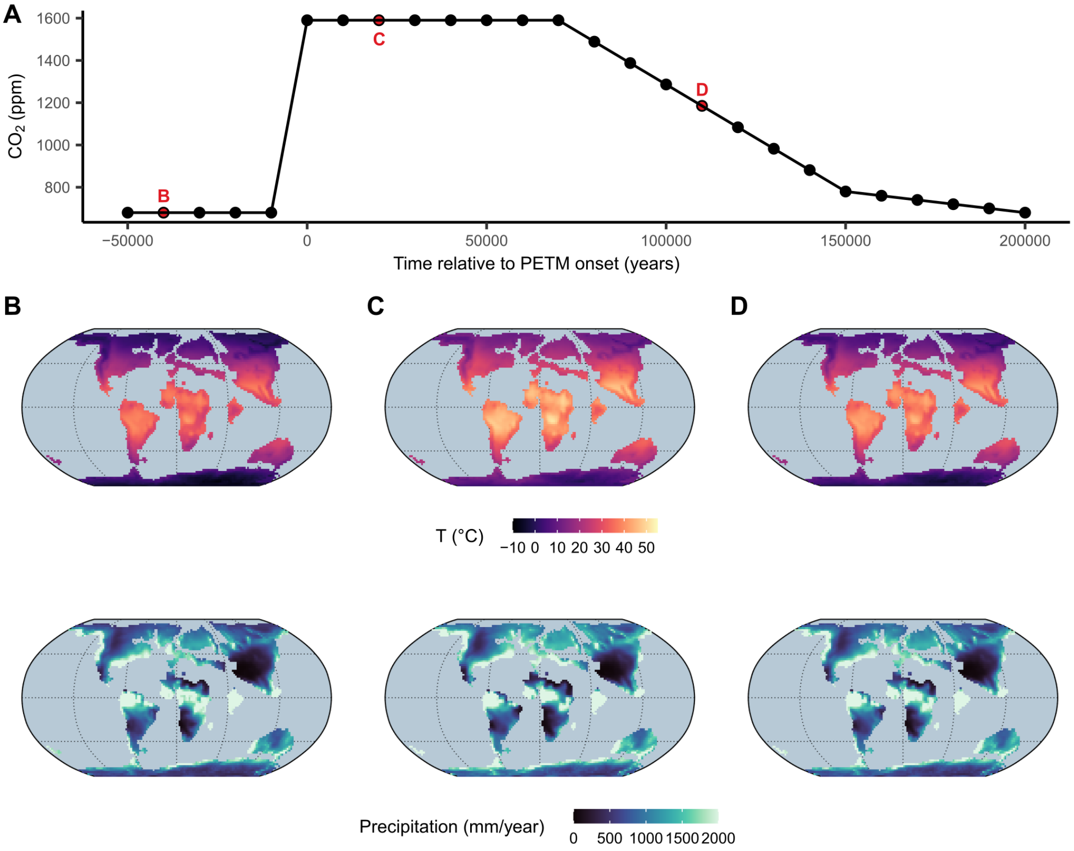

Supplementary Figure 7 Community Earth System Model (CESM) 1.2 Paleocene–Ecoene Thermal Maximum (PETM) climate boundary conditions considered for the vegetation modelling. **A.** CO_2 curve considered in the study. **B.**, **C.** and **D.** represent annual temperature (T, top row) and precipitation averages (bottom row) for pre-PETM conditions (680 ppm), the PETM body (1590 ppm), and the middle of the PETM recovery period, respectively. Figure produced using R [3] and the ggplot2 package [4].

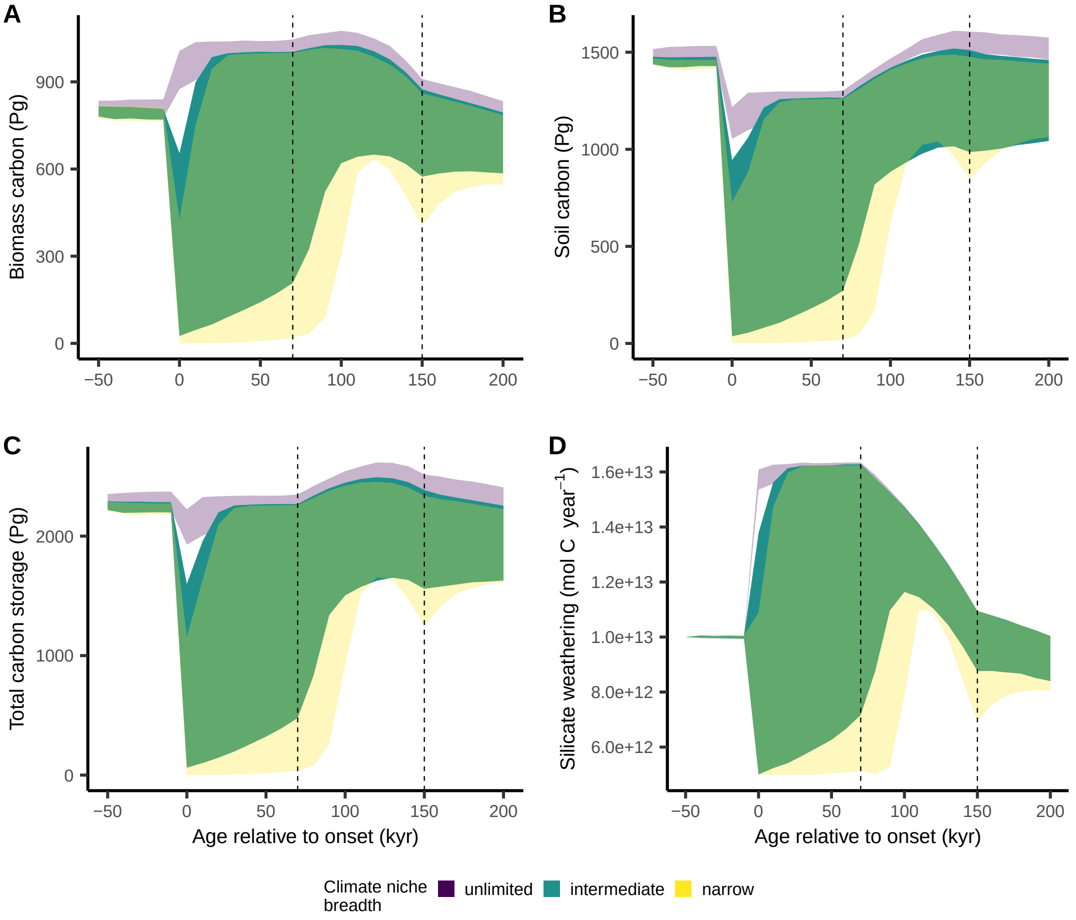

Supplementary Figure 8 Vegetation-mediated carbon cycling for different climatic niche breadths of modelled plants. A. Modelled biomass carbon storage (aboveground + belowground). **B.** Soil carbon storage as a function of carbon inputs from net primary productivity and temperature-dependent heterotrophic carbon respiration. **C.** Total carbon storage in biomass and soil. **D.** Silicate weathering as a function of erosion, runoff, temperature and vegetation-mediated weathering enhancement. Coloured areas give the range of modelled trajectories observed under different climatic niche breadth scenarios and considering different rates of trait evolution (α between 0.01 and 1) and dispersal (scale of dispersal kernel between 200 and 1100 km per 10^4 years) of modelled plants. The default model ("intermediate") considers a maximum of 10°C of temperature deviation from a plant's adapted mean annual temperature, warmest month temperature, and coldest month temperature before a loss of productivity occurs. The "narrow" climatic niche breadth considers a maximum 5°C deviation, while the unlimited model considers modelled plants to have no thermal tolerance limits. Figure produced using R [3] and the ggplot2 package [4].

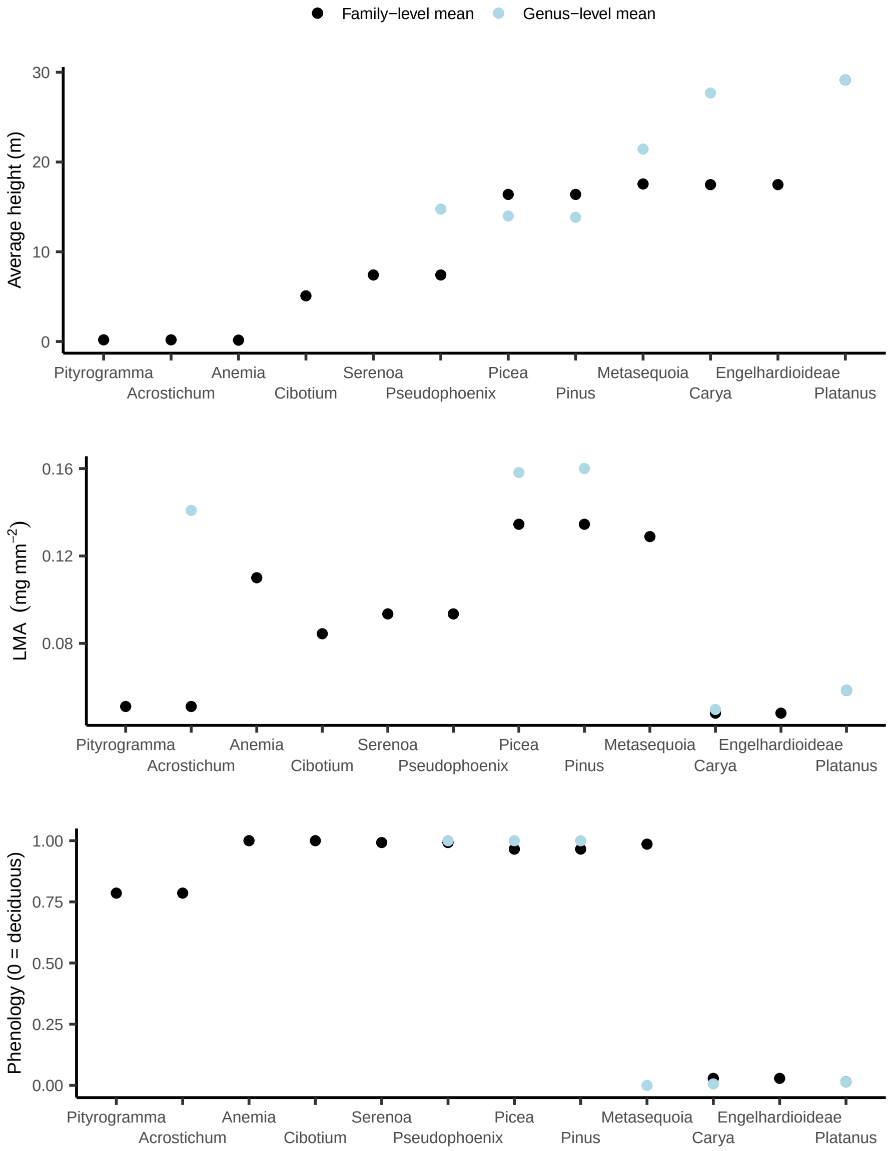

Supplementary Figure 9 Selected nearest living relative trait values. Sample trait values of height, leaf mass per area (LMA) and phenology for important taxa. Assigned trait values represent the average value across present-day observations of the same family or genus documented in the plant trait data base TRY. Each observation in the TRY data base is given equal weight, and the average will approximate the most common trait value within a family or genus. Figure produced using R [3] and the ggplot2 package [4].

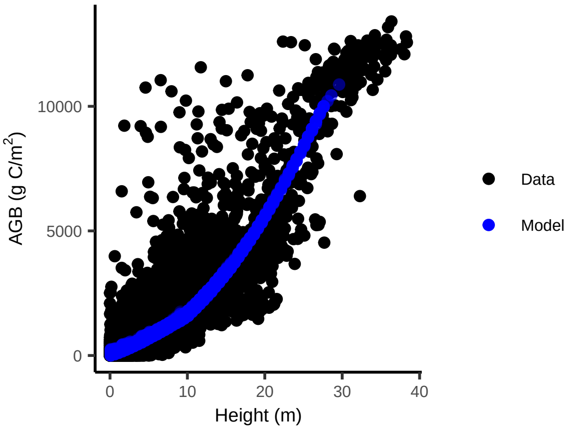

Supplementary Figure 10 Relationship between vegetation height and aboveground biomass carbon density (AGB). Data is derived from a satellite- and machine-learning-based vegetation height estimate for the year 2020 [5] and a machine-learning-based upscaling approach of field measurements for biomass [8]. Blue points represent the height to biomass relationship observed in the Trait Ecology and Evolution vegetation model over Deep time (TREED) for present-day climatic and geographic boundary conditions. Figure produced using R [3] and the ggplot2 package [4].

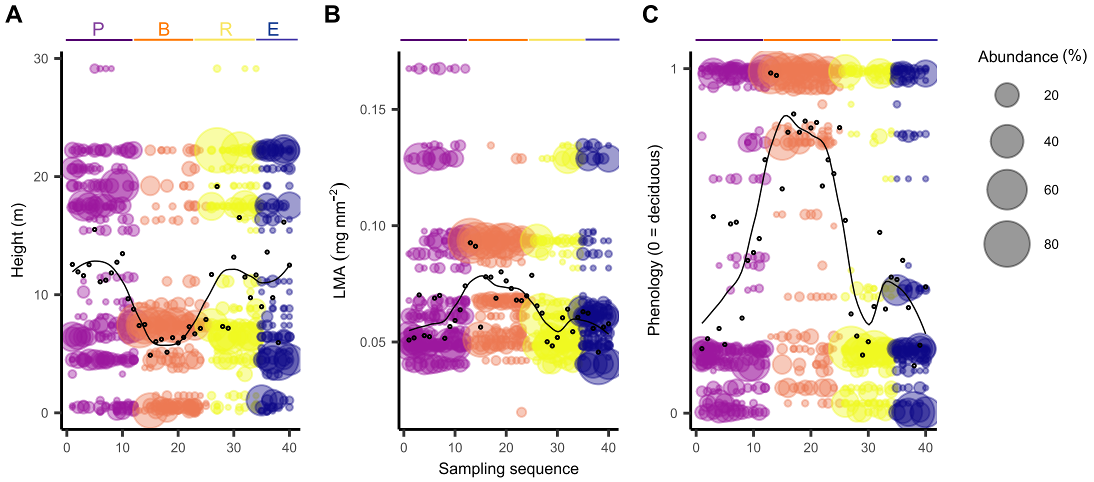

Supplementary Figure 11 Vegetation trait reconstruction across the Paleocene-Eocene Thermal Maximum (PETM) using a nearest living relative approach for the Bighorn Basin sampling site. Samples are ordered in sequence and binned into time bins of the Paleocene (P), the PETM body (B), the PETM recovery (R) and the Eocene (E). Trait values were assigned to the fossil species observed in the sediment cores based on present-day trait values of species in the same family. The position and size of the points indicate the assigned trait value and the abundance of the fossil species in the sample. Black points indicate the abundance-weighted mean per sample; the black line is a generalized additive model (GAM) smoother of the abundance-weighted means over time. The considered traits are: **A.** plant height, **B.** leaf mass per area (LMA), and **C.** phenology (deciduous or evergreen). Figure produced using R [3] and the ggplot2 package [4].

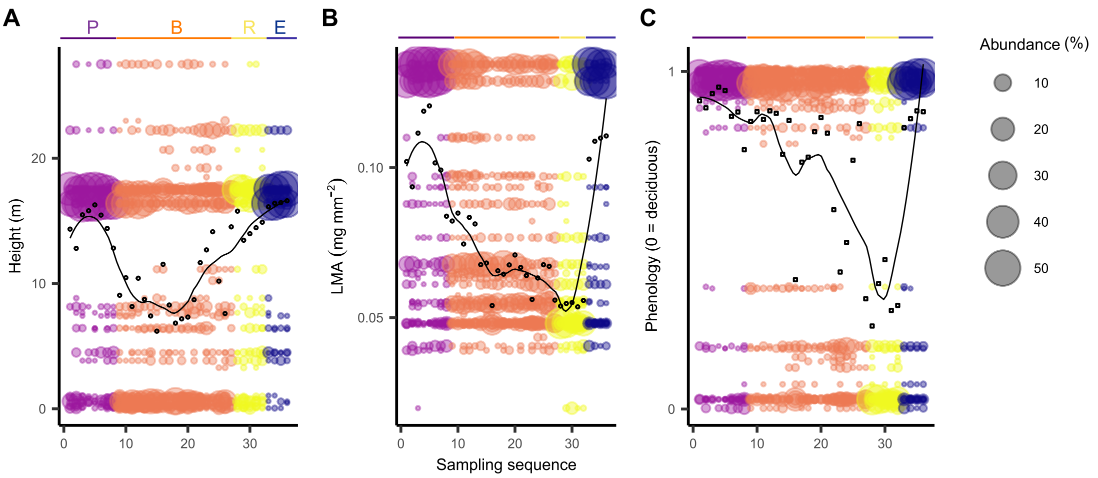

Supplementary Figure 12 Vegetation trait reconstruction across the Paleocene-Eocene Thermal Maximum (PETM) using a nearest living relative approach for the 22/11-N sampling site. Samples are ordered in sequence and binned into time bins of the Paleocene (P), the PETM body (B), the PETM recovery (R) and the Eocene (E). Trait values were assigned to the fossil species observed in the sediment cores based on present-day trait values of species in the same family. The position and size of the points indicate the assigned trait value and the abundance of the fossil species in the sample. Black points indicate the abundance-weighted mean per sample; the black line is a generalized additive model (GAM) smoother of the abundance-weighted means over time. The considered traits are: **A.** plant height, **B.** leaf mass per area (LMA), and **C.** phenology (deciduous or evergreen). Figure produced using R [3] and the ggplot2 package [4].

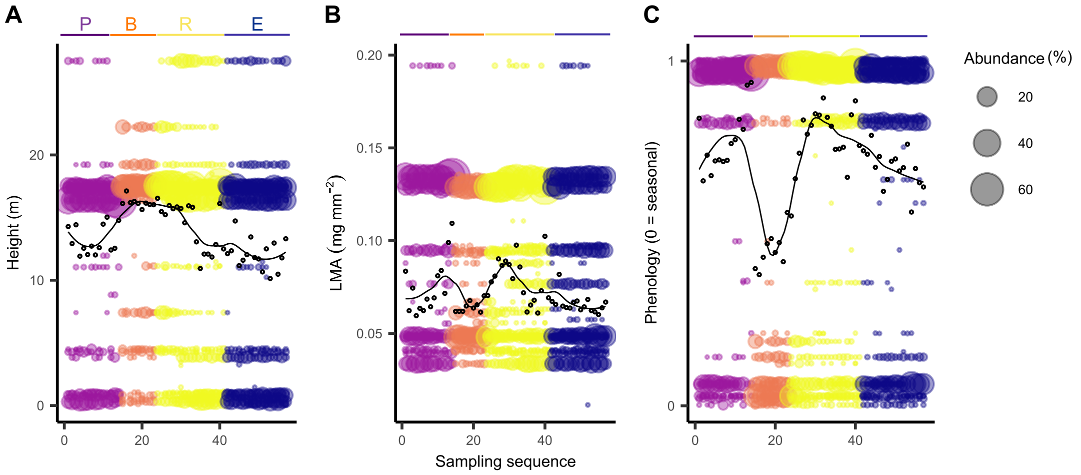

Supplementary Figure 13 Vegetation trait reconstruction across the Paleocene-Eocene Thermal Maximum (PETM) using a nearest living relative approach for the ACEX (Arctic Coring Expedition) sampling site. Samples are ordered in sequence and binned into time bins of the Paleocene (P), the PETM body (B), the PETM recovery (R) and the Eocene (E). Trait values were assigned to the fossil species observed in the sediment cores based on present-day trait values of species in the same family. The position and size of the points indicate the assigned trait value and the abundance of the fossil species in the sample. Black points indicate the abundance-weighted mean per sample; the black line is a generalized additive model (GAM) smoother of the abundance-weighted means over time. The considered traits are: **A.** plant height, **B.** leaf mass per area (LMA), and **C.** phenology (deciduous or evergreen). Figure produced using R [3] and the ggplot2 package [4].

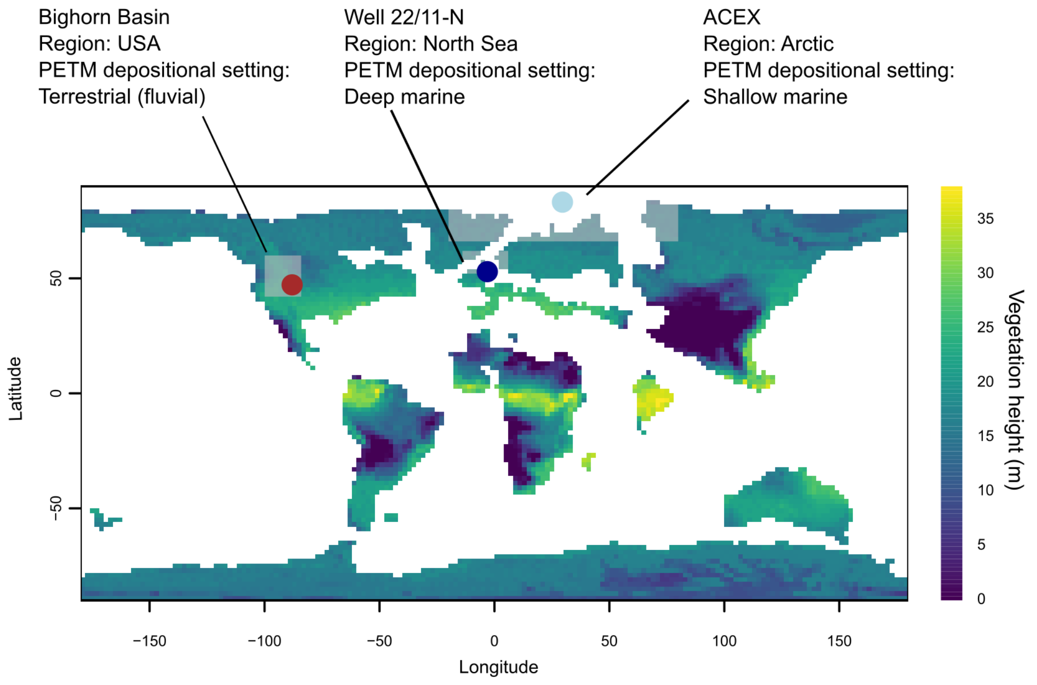

Supplementary Figure 14 Locations of sampling sites and considered model regions. Coloured circles indicate the paleolocations of the considered sampling sites. The grey-shaded areas indicate the considered regions of the model for the model–data comparison of vegetation structures across the Paleocene–Eocene Thermal Maximum (PETM). The background map displays the late Paleocene vegetation height as derived from the vegetation model. Figure produced using R [3] and the raster package [13].

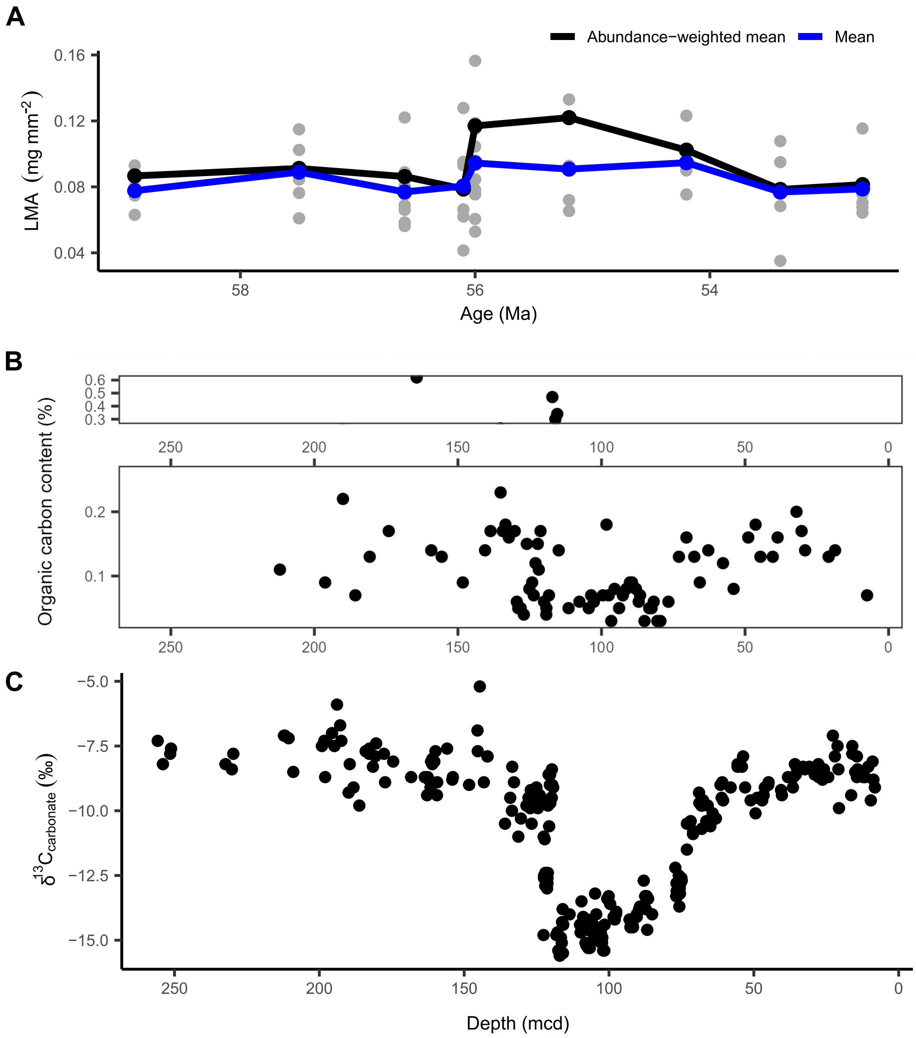

Supplementary Figure 15 Macrofossil and soil observations from the Bighorn Basin from the late Paleocene to early Eocene. A. Petiole width-based estimates of leaf mass per area (LMA) for dicot angiosperms in the Bighorn Basin between the late Paleocene and early Eocene, including the Paleocene–Eocene Thermal Maximum (PETM) at 56 Ma, from [14, 15]. **B.** Organic carbon contents from the Bighorn Basin Coring Project (BBCP) core drilled at Polecat Bench (Bighorn Basin). **C.** δ^{13}C of paleosol carbonates from the BBCP core indicating the PETM location with a negative isotope excursion. Figure produced using R [3] and the ggplot2 package [4].

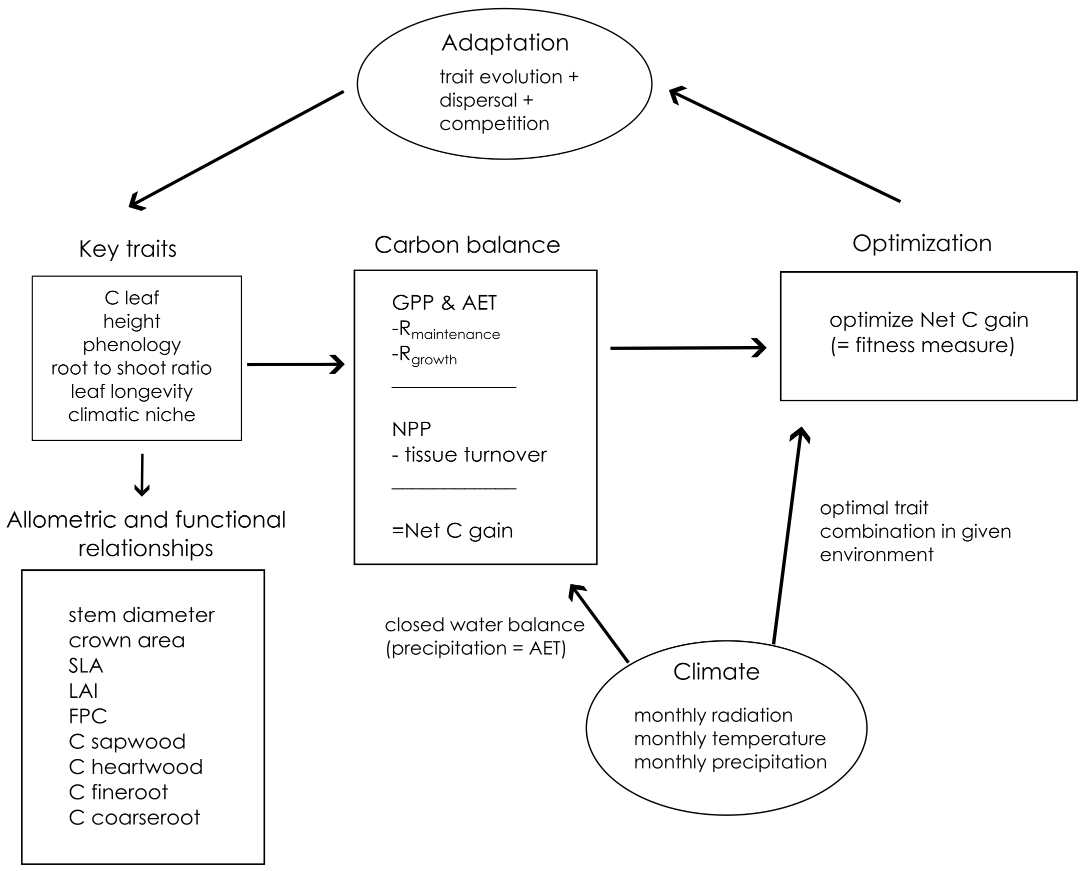

Supplementary Figure 16 Schematic overview of the model processes. Abbreviations: C = carbon, SLA = specific leaf area, LAI = leaf area index, FPC = foliage projective cover, GPP = gross primary productivity, NPP = net primary productivity, AET = actual evapotranspiration, $R_{maintenance}$ = maintenance respiration, R_{growth} = growth respiration, NPP = net primary productivity

Supplementary Methods

Extended vegetation model description

The TREED vegetation model (version 0.1) is initialized with one reference model plant per terrestrial grid cell. The trait combination of the occupying reference plant is considered to represent the trait combination of the dominating vegetation type at a location. The model consists of four modules that are executed in succession, following the scheme in Supplementary Fig. 16.

Allometric and functional trait relationships

Every modelled plant is characterized by six key traits, including the size of the leaf carbon pool (C_{leaf}), height (H), phenology (deciduous or evergreen habit), a fine root to leaf carbon ratio ($r : s$), leaf longevity (a_{ll}) and a climatic niche that describes a plant's bio-climatic limits for productivity. Based on these key traits and using allometric and functional trait relationships commonly used in dynamic vegetation models [16, 17], a number of other plant characteristics are approximated. Following the pipe model [18, 19], leaf area (LA) and sapwood cross-section area (SA) are assumed proportional (all model parameters and variables, including units, are listed in Supplementary Tables 1 and 2):

$$\mathrm{LA} = k_{\mathrm{la:sa}} \cdot \mathrm{SA} \tag{1}$$

Leaf and fine root carbon allocation (C_{fineroot}) are related by the plant's $r : s$ trait:

$$C_{\mathrm{leaf}} = r : s \cdot C_{\mathrm{fineroot}} \tag{2}$$

Following [20], plant height H and plant diameter D are assumed to relate according to:

$$H = k_{\mathrm{allom2}} \cdot D^{k_{\mathrm{allom3}}} \tag{3}$$

With increasing height, a plant's crown area (CA), i.e., the surface area occupied by the plant, is assumed to increase following:

$$\mathrm{CA} = \min(k_{\mathrm{allom1}} \cdot D^{k_{\mathrm{rp}}}, \mathrm{CA}_{\mathrm{max}}) \tag{4}$$

with a maximum crown area (CA_{max}) of $15m^2$ [16].

Specific leaf area (SLA) is related to leaf longevity (a_{ll}), representing a carbon-economic trade-off between short leaf longevity (higher construction costs) and high SLA (higher carbon capture potential) [11]. The consideration of a continuous relationship between leaf longevity and SLA is a key difference to PFT-based vegetation models, where different vegetation types are assigned fixed values of a_{ll} and SLA. In the current model, these variables can evolve in response to environmental changes, as explained later. The generalized relationship represents an intermediate form as considered as basis for evergreen and deciduous plants in the LPJml4 model [17]:

$$\mathrm{SLA} = (2 \cdot 10^{-4}) \cdot \frac{1}{\mathrm{DM}_c} \cdot 10^{2.25-0.5\cdot\log(a_{ll}\cdot 12)} \tag{5}$$

where DM_c represents an assumed average dry matter carbon content of 0.47 g C per g of dry matter.

A plant's leaf area index (LAI) can then be calculated as:

$$\mathrm{LAI} = \frac{C_{\mathrm{leaf}} \cdot \mathrm{SLA}}{\mathrm{CA}} \tag{6}$$

Using the Lambert-Beer law, the ground covered by leaves (foliage projective cover; FPC) is derived from the LAI using a light extinction coefficient (k) of 0.6 for deciduous plants with leaf longevities of a_{ll} below 1 year (high SLA), 0.4 for plants with leaf longevities larger than 4 years (low SLA) and 0.5 in all other cases:

$$\mathrm{FPC} = 1 - e^{-k\cdot\mathrm{LAI}} \tag{7}$$

Sapwood carbon is derived from the plant SA and H and assuming a cylindrical geometry and a constant wood density (WD):

$$C_{\mathrm{sapwood}} = \mathrm{SA} \cdot H \cdot \mathrm{WD} \tag{8}$$

A sapwood carbon pool is calculated for all plant heights, accounting for structural carbon in the case of small plants that would not be considered trees. Considering woody tissue as the by far most important component of global above ground biomass [21], a power law derived from present-day canopy height [5] and biomass density [8] describes the relationship between modelled reference plant volume ($H \cdot \mathrm{CA}$) and total stem biomass (sapwood and heartwood

carbon; $C_{\text{sapwood}} + C_{\text{heartwood}}$):

$$C_{\text{stem}} = k_{\text{intercept}} \cdot (\text{CA} \cdot H)^{k_{\text{pow}}} \tag{9}$$

Coarse root carbon ($C_{\text{coarseroot}}$) is assumed to be ≈ 0.25 of the stem biomass ($C_{\text{heartwood}}$ and C_{sapwood}), as derived from [8], considering variation depending on the plant's $r : s$ trait:

$$C_{\text{coarseroot}} = 0.25 \cdot (C_{\text{heartwood}} + C_{\text{sapwood}}) \cdot r : s \tag{10}$$

All allometric constants (k_{allom1}, k_{allom2}, k_{allom3}, k_{rp}, $k_{\text{la:sa}}$, $k_{\text{intercept}}$, k_{pow}) are calibrated using present-day canopy height and aboveground biomass data.

Carbon balance

The traits and the derived plant characteristics determine the capacity of plants to capture carbon through photosynthesis, as well as the carbon costs associated with maintenance respiration, growth respiration and tissue turnover.

Following [17], photosynthesis is modelled based on the Farquhar photosynthesis model [22, 23], considering generalizations for global modelling applications by [24]. The "strong optimality" hypothesis [25] is applied, assuming that nitrogen content and Rubisco activity of leaves vary seasonally and with canopy position in a way to maximize net assimilation at the leaf level. The resulting model has the form of a "light-use efficiency" model, considering photosynthetically active radiation (PAR), temperature, day length, water availability and canopy conductance as modulating factors of the photosynthetic capacity.

The absorbed photosynthetically active radiation (APAR) is calculated as a fraction of PAR, considering a grid cell's FPC (eq. 7), a leaf to stand level scaling factor $\alpha_{\text{leaf:stand}}$, and a climatic niche suitability factor, which reduces the light absorption potential for modelled plants under unsuited climatic conditions:

$$\text{APAR} = \text{PAR} \cdot \text{FPC} \cdot \alpha_{\text{leaf:stand}} \cdot \Phi_{\text{nichestress}} \tag{11}$$

Half of the surface incoming solar radiation is considered PAR ($\text{PAR} = 0.5 \cdot \text{RDSD}$, where RSDS is the downwelling surface shortwave radiation). The climatic niche stress factor $\Phi_{\text{nichestress}}$ accounts for temperature deviations between the local environment and the temperature distribution to which the occurring vegetation is adapted. The climatic niche stress factor can reduce

the vegetation's carbon capture potential after the dispersal into new environments, or following abrupt changes in local climatic conditions. As part of the trait set of the modelled reference plants, the climatic niche is characterized by $T_{\mathrm{min,veg}}$, $T_{\mathrm{max,veg}}$ and $T_{\mathrm{mean,veg}}$, representing the average coldest and warmest month temperature and the mean annual temperature of the environment to which the represented vegetation is best adapted, respectively. If the local warmest monthly temperature exceeds the $T_{\mathrm{max,veg}}$ or if the coldest monthly temperature is below the $T_{\mathrm{min,veg}}$, a heat and a cold stress factor is calculated (Φ_{heat}, Φ_{cold}, respectively). Similarly, it results in a biotic stress if there is a large deviation between the local mean annual temperature and the mean temperature to which the modelled reference plant is adapted (Φ_{mean}):

$$\Phi_{\mathrm{heat}} = \exp(-k_{\mathrm{nichebreadth}} \cdot (T_{\mathrm{max,local}} - T_{\mathrm{max,veg}})^2) \tag{12}$$

$$\Phi_{\mathrm{cold}} = \exp(-k_{\mathrm{nichebreadth}} \cdot (T_{\mathrm{min,local}} - T_{\mathrm{min,veg}})^2) \tag{13}$$

$$\Phi_{\mathrm{mean}} = \exp(-k_{\mathrm{nichebreadth}} \cdot (T_{\mathrm{mean,local}} - T_{\mathrm{mean,veg}})^2) \tag{14}$$

The $k_{\mathrm{nichebreadth}}$ parameter describes the impact of a deviation in temperatures from the vegetation's climatic niche. $k_{\mathrm{nichebreadth}}$ can be considered a description of the size of the fundamental niche of the modelled reference plants. In the current model set up, three different $k_{\mathrm{nichebreadth}}$ parameters can be tested: 0, 0.03 and 0.1, describing an unlimited, an intermediate and a narrow fundamental climatic niche breadth. As a reference, $k_{\mathrm{nichebreadth}} = 0.03$ allows for a maximum 10°C deviation of the coldest or warmest month temperature, or the mean annual temperature from $T_{\mathrm{min,veg}}$, $T_{\mathrm{max,veg}}$ or $T_{\mathrm{mean,veg}}$, respectively, until a loss of productivity occurs. The overall stress index $\Phi_{\mathrm{nichestress}}$ is defined as

$$\Phi_{\mathrm{nichestress}} = \min(\Phi_{\mathrm{heat}}, \Phi_{\mathrm{cold}}, \Phi_{\mathrm{mean}}) \tag{15}$$

The climatic niches of the modelled reference plants are not fixed throughout the simulation. It is assumed that in response to natural selection $T_{\mathrm{max,veg}}$, $T_{\mathrm{min,veg}}$ and $T_{\mathrm{mean,veg}}$ of the reference plant steadily evolve towards local climatic conditions. Thus, the longer a reference plant persists in a given environment, the higher the degree of adaptation to the local climate. The rate of climatic niche evolution is a user-defined model parameter, described in more detail below.

Based on APAR, photosynthesis is then calculated as the minimum of light-limited (J_E) and Rubisco-limited photosynthesis (J_C) [25]. Light-limited photosynthesis is computed as:

$$J_E = C_1 \cdot \frac{\text{APAR}}{\text{daylength}} \tag{16}$$

with

$$C_1 = \alpha_{C_3} \cdot f_{\text{temp}} \cdot \left(\frac{p_i - \Gamma_*}{p_i + 2 \cdot \Gamma_*} \right) \tag{17}$$

p_i is the leaf internal partial pressure of CO_2. p_i is given by $p_i = \lambda \cdot p_a$, where p_a is the ambient partial pressure of CO_2 and λ (between 0–0.8) describes the plant-atmosphere water and carbon exchange, depending on the plant's water status and stomatal opening. α_{C_3} is the intrinsic quantum efficiency for CO_2 uptake. f_{temp} describes the general dependency of the efficiency of the photosynthetic pathway to temperature, adopted from [17] and generalized for all plants as:

$$\begin{aligned} f_{\text{temp,low}} &= \frac{1}{(1+\exp(0.25 \cdot (12-T))} \\ f_{\text{temp,high}} &= 1 - \frac{1}{1+\exp(-(T-40.85))} \\ f_{\text{temp}} &= f_{\text{temp,low}} \cdot f_{\text{temp,high}} \end{aligned} \tag{18}$$

with an inhibition of photosynthesis for monthly average temperatures below 0°C and higher than 45°C, and an optimum photosynthetic rate between 25–30°C. Finally, Γ_* in eq. (17) represents the photorespiratory CO_2 compensation point:

$$\Gamma_* = \frac{[O_2]}{2 \cdot \tau} \tag{19}$$

where $\tau = \tau_{25} \cdot q_{10_\tau}^{\frac{T-25}{10}}$ is the specificity factor that reflects the ability of Rubisco to discriminate between CO_2 and O_2. $[O_2]$ is the partial pressure of O_2, τ_{25} the τ value at 25°C, and q_{10_τ} a temperature sensitivity parameter.

The Rubisco-limited photosynthesis J_C is calculated as

$$J_C = C_2 \cdot V_m \tag{20}$$

with

$$C_2 = \frac{p_i - \Gamma_*}{p_i + K_C \left(1 + \frac{[O_2]}{K_O}\right)} \tag{21}$$

where K_C and K_O are Michaelis-Menten constants for CO_2 and O_2, respectively. V_m represents the maximum Rubisco capacity, calculated as

$$V_m = \frac{1}{b} \cdot \frac{C_1}{C_2} \cdot ((2 \cdot \theta - 1) \cdot s - (2 \cdot \theta \cdot s - C_2) \cdot \sigma) \cdot \mathrm{APAR} \tag{22}$$

where b is a static leaf respiration coefficient, θ a shape parameter that describes the co-limitation of light and Rubisco activity [25] and

$$\sigma = \sqrt{1 - \frac{C_2 - s}{C_2 - \theta \cdot s}} \tag{23}$$

and

$$s = \frac{24}{\mathrm{daylength}} \cdot b \tag{24}$$

V_m is calculated using the maximum leaf-atmosphere carbon and water exchange parameter $\lambda = \lambda_{max}$.

Daily gross photosynthesis (A_{gd}) is calculated as

$$A_{\mathrm{gd}} = \frac{\left(J_E + J_C - \sqrt{(J_E + J_C)^2 - 4 \cdot \theta \cdot J_E \cdot J_C}\right)}{2 \cdot \theta} \cdot \mathrm{daylength} \tag{25}$$

Subtracting the daytime leaf respiration, the daily net daytime photosynthesis (A_{dt}) is derived.

$$A_{\mathrm{dt}} = A_{\mathrm{gd}} - \left(\frac{\mathrm{daylength}}{24}\right) \cdot R_{\mathrm{leaf}} \tag{26}$$

Thereby, dark respiration of leaves depends on the maximum Rubisco capacity following:

$$R_{\mathrm{leaf}} = V_m \cdot b \tag{27}$$

The photosynthetic rate relates to the canopy conductance for water vapor (g_c) through the CO_2 diffusion gradient between the intercellular airspace and the atmosphere, according to [17]:

$$g_c = \frac{1.6 \cdot A_{\mathrm{dt}}}{p_a \cdot (1 - \lambda)} + g_{\mathrm{min}} \tag{28}$$

where g_{min} is a minimum canopy conductance that occurs due to non-photosynthesis related processes. The canopy conductance depends on the local water availability and the atmospheric water demand. The exchange of water and carbon with the atmosphere is controlled

by the parameter λ, with a maximum λ of 0.8 indicating non-water limited conditions and a maximum water and carbon exchange, whereas low λ values indicate stomatal closure and limited water and carbon exchange. A potential water limitation is evaluated by comparing the atmospheric water demand (E_{demand}) and the supply of water, approximated by the monthly average precipitation rate (E_{supply}). Following [17] and [26], E_{demand} is calculated as:

$$E_{\text{demand}} = E_{\text{eq}} \cdot \frac{\alpha_m}{1 + \frac{g_m}{g_c}} \tag{29}$$

employing a Priestley-Taylor coefficient of $\alpha_m = 1.391$ and a conductance scaling factor $g_m = 3.26$ mm/s. E_{eq} is the equilibrium evapotranspiration rate calculated as:

$$E_{\text{eq}} = \frac{\text{sa}}{\text{sa} + \gamma} \cdot \frac{R_n}{\lambda_{\text{vap}}} \tag{30}$$

with sa being the slope of the saturation vapor pressure curve, γ the psychrometric constant, and R_n the daytime net radiation as derived from the climate inputs.

For calculating photosynthesis and evapotranspiration rates in cases where $\lambda = \lambda_{\text{max}}$ results in $E_{\text{demand}} > E_{\text{supply}}$, a bisection algorithm is employed to find a canopy-atmosphere gas exchange parameter λ that satisfies eqs. (26) and (28), and that results in a closed water balance ($E_{\text{demand}} = E_{\text{supply}}$), solving for:

$$0 = A_{\text{dt}} - A_{\text{dt}} = A_{\text{gd}} - \left(\frac{\text{daylength}}{24}\right) \cdot R_{\text{leaf}} - p_a \cdot (g_c - g_{\text{min}}) \cdot \frac{1 - \lambda}{1.6} \tag{31}$$

and the canopy conductance and photosynthetic rates are recalculated with the obtained λ that results in a closed water balance.

Following [16], actual evapotranspiration (AET) is subsequently calculated as

$$\text{AET} = \min(E_{\text{supply}}, E_{\text{demand}}) \tag{32}$$

Besides the leaf carbon pool, the sapwood and fine root carbon pools are associated with maintenance respiration that depend on tissue-specific C:N ratios, calculated as:

$$R_{\text{sapwood}} = r \cdot \text{AF} \cdot \frac{C_{\text{sapwood}}}{\text{C : N}_{\text{wood}}} \cdot g(T_{\text{air}}) \cdot \frac{1}{\text{CA}} \tag{33}$$

$$R_{\text{fineroot}} = r \cdot \text{AF} \cdot \frac{C_{\text{fineroot}}}{\text{C : N}_{\text{root}}} \cdot g(T_{\text{soil}}) \cdot \frac{1}{\text{CA}} \tag{34}$$

where r is a base respiration rate, $\text{C : N}_{\text{wood}}$ and $\text{C : N}_{\text{root}}$ are the C:N ratios in wood and root tissue, respectively. AF represents an acclimation factor that accounts for the acclimation of respiration to lower rates under warmer temperatures [27]. AF is 1 for temperatures below 10°C, 0.3 for temperatures higher than 30°C and

$$\text{AF} = 1.35 - 0.035 \cdot T \tag{35}$$

for temperatures in between. $g(T_{\text{air}})$ and $g(T_{\text{soil}})$ describe the temperature dependency of respiration following [28]:

$$g(T) = \exp\left(308.56 \cdot \left(\frac{1}{56.02} - \frac{1}{T + 46.02}\right)\right) \tag{36}$$

with T either being the air or the soil temperature.

Using monthly average climate data (temperature, precipitation, radiation), carbon fluxes are calculated as monthly averages, and are integrated over the course of the year. For deciduous, summergreen plants, fluxes are integrated over the warmest months of the year up to the duration of the leaf longevity, that is assumed to limit the growing season length. The annual net primary productivity (NPP) is then calculated as:

$$\text{NPP} = (1 - r_{\text{gr}}) \cdot (\text{GPP} - R_{\text{leaf}} - R_{\text{sapwood}} - R_{\text{fineroot}}) \tag{37}$$

where r_{gr} accounts for a fixed fraction of carbon assumed to be invested for growth respiration following [17], and GPP is the annual sum of the monthly calculated rates of average daytime gross photosynthesis (A_{gd}).

Using the calculated NPP rates, soil carbon stocks are approximated based on a reduced complexity soil carbon model from [29]. The model represents a one dimensional soil layer at steady state, considering carbon inputs from NPP and heterotrophic soil carbon respiration. It is assumed that soil heterotrophic respiration is directly proportional to the soil carbon pool and modulated by temperature according to a Q_{10} function with baseline temperature 15°C [28]:

$$C_{\text{soil}} = \frac{\text{NPP}}{k \cdot Q_{10}^{(T-15)/10}} \tag{38}$$

with a uniform k of $\frac{1}{16}$ year^{-1}, Q_{10} of 1.75 and T being the annual average temperature.

With only one reference plant per grid cell, the model does not explicitly represent establishment, growth and mortality of plant individuals in a location. Instead, in order to evaluate whether a certain growth form is appropriate for a location, an annual average carbon balance is calculated. To do so, annual average tissue turnover carbon costs are estimated based on the reference plant's characteristics and size of the carbon pools. The carbon balance ensures that the average carbon capture potential of the vegetation represented by the reference plant is sufficient to build and maintain the modelled vegetation characteristics at a location. The calculated yearly tissue turnover of sapwood, heartwood and coarse root are calculated as:

$$\tau_{\text{sapwood}} = C_{\text{sapwood}} \cdot f_{\text{sapwood}} \cdot (1 + \text{HD} + \text{FD}) \cdot \frac{1}{\text{CA}} \tag{39}$$

$$\tau_{\text{heartwood}} = C_{\text{heartwood}} \cdot f_{\text{heartwood}} \cdot (1 + \text{HD} + \text{FD}) \cdot \frac{1}{\text{CA}} \tag{40}$$

$$\tau_{\text{coarseroot}} = C_{\text{coarseroot}} \cdot f_{\text{coarseroot}} \cdot (1 + \text{HD} + \text{FD}) \cdot \frac{1}{\text{CA}} \tag{41}$$

where f represents a tissue-specific turnover time and HD and FD account for increased tissue turnover (maximum doubling) in extreme heat and frost environments, respectively. They are calculated as:

$$\text{HD} = 2 \cdot \frac{\min(M_{\text{heat}}, 5)}{5} \tag{42}$$

$$\text{FD} = 2 \cdot \frac{\min(M_{\text{frost}}, 5)}{5} \tag{43}$$

where M_{heat} is the number of months with average temperatures larger than 50°C and M_{frost} is the number of months with average temperatures lower than −20°C for evergreens and −10°C for deciduous plants, accounting for the tendency of increased frost tolerance for evergreen plants [17]. The fine root and leaf carbon turnover times depend on the plant's phenology. Annual leaf carbon building costs relate to the leaf longevity (in years),

$$\tau_{\text{leaf}} = \begin{cases} C_{\text{leaf}} \cdot \frac{1}{a_{\text{ll}}} \cdot \frac{1}{\text{CA}} & \text{evergreens} \\ C_{\text{leaf}} \cdot 1 \cdot \frac{1}{\text{CA}} & \text{deciduous} \end{cases} \tag{44}$$

with the C_{leaf} renewed once per year for deciduous plants. For leaf longevities longer than a year, leaf and fine root carbon turnover are assumed to be proportional [17]:

$$\tau_{\text{fineroot}} = \begin{cases} C_{\text{fineroot}} \cdot \frac{1}{a_{\text{ll}}} \cdot \frac{1}{\text{CA}} & a_{\text{ll}} > 1 \\ C_{\text{fineroot}} \cdot 1 \cdot \frac{1}{\text{CA}} & a_{\text{ll}} \leq 1 \end{cases} \tag{45}$$

The tissue carbon turnover costs are important for determining the biomass that can be sustained in a given environment and to determine whether a deciduous or an evergreen phenology results in a more favorable (positive) carbon balance. For the calculation of the annual carbon balance, all carbon and water fluxes (except for GPP, as already given as rate per area) associated with the different carbon pools are area-normalized using the modelled plant's crown area. Thereby, fluxes calculated for the reference plant are assumed to apply to the entire grid cell area. Together, the modelled plant's annual carbon balance can be written as:

$$\text{NCG} = \text{NPP} - \tau_{\text{leaf}} - \tau_{\text{sapwood}} - \tau_{\text{heartwood}} - \tau_{\text{coarseroot}} - \tau_{\text{fineroot}} \tag{46}$$

where NCG is the net carbon gain that represents the carbon that is available to the modelled reference plants after accounting for maintenance and growth respiration, as well as carbon investments for tissue building. The NCG is assumed to be invested into reproduction, defense, nutrient acquisition, symbiotic relationships, stress tolerance and other processes relevant to survival. As such, it is considered a fitness measure of a modelled plant in a given environment.

Trait optimization

The plant traits that characterize the vegetation in each grid cell of the model do not remain constant throughout the simulation, but are subject to adaptation processes and evolution. Based on optimality principles [30, 31], it is assumed that natural selection will result in average trait combinations evolving towards increasingly optimal adaptation to local environmental conditions with time. In order to imitate this selection process, a prediction of the optimal trait combination in any given environment is needed. At each time step and for every grid cell of the model, this prediction is obtained by applying an optimization algorithm that predicts an optimum trait combination (considering C_{leaf}, phenology, a_{ll}) that maximizes the NCG under given environmental conditions of solar radiation, temperature, and precipitation. Optionally, the fine root to leaf carbon ratio ($r : s$) can also be optimized, considering a trade-off with water availability. However, the consideration of the additional optimization parameter requires an increase in number of iterations in the optimization algorithm. For simplicity and to reduce computational cost, $r : s$ is assumed fixed at 1 in the default model configuration. For the optimization, a differential evolution algorithm for multi-parameter global optimizations 'DEoptim' is employed [32]. In environments with more than 3 months with average temperatures below 3°C (necessary condition for deciduous phenology), the optimization procedure further evaluates whether a deciduous or an evergreen phenology results in a larger NCG.

Additionally, in this model step, the maximum potential height of a grid cell's reference plants is evaluated. In the model, it is generally assumed that light competition will favor tall plants over short plants so that evolution will result in the vegetation canopy evolving towards the assessed maximum potential height. The potential height is limited by the height-associated increase of carbon costs for tissue respiration and turnover [33]. The maximum potential height is defined as the height at which the ratio of NCG/NPP is approximately 20%. This minimum NCG is assumed necessary to ensure reproductive success.

Adaptation

At every time step, the traits of the modelled plants will evolve towards the predicted optimum trait combination in a given environment. The rate of evolutionary trait adaptation is a user-defined adaptation rate α. α is a unitless fraction, resulting in larger changes for larger deviations of current traits from the optimal trait values (i.e., representing increased selection pressure). An α value of 0 represents highly conserved traits, whereas 1 represent a near-immediate adaptation. Adaptation is considered to represent a range of adaptation processes that include acclimation, phenotypic plasticity, changes in vegetation composition that could change average trait combinations and finally, adaptive evolutionary processes that are particularly relevant on long, geologic timescales. Further, to allow a limited degree stochasticity in the evolutionary adaptation in space and time, $\mathcal{N}(\alpha, 0.05)$ indicates that the rate of evolution is drawn from a normal distribution with mean α as defined for the simulation run and a standard deviation of 0.05. Together, trait adaptation is computed as:

$$C_{\text{leaf,new}} = C_{\text{leaf,old}} + \mathcal{N}(\alpha, 0.05) \cdot (C_{\text{leaf,target}} - C_{\text{leaf,old}}) \tag{47}$$

$$a_{\text{ll,new}} = a_{\text{ll,old}} + \mathcal{N}(\alpha, 0.05) \cdot (a_{\text{ll,target}} - a_{\text{ll,old}}) \tag{48}$$

$$H_{\text{new}} = H_{\text{old}} + \mathcal{N}(\alpha, 0.05) \cdot (H_{\text{potential}} - H_{\text{old}}) \tag{49}$$

In the current model version H, is assumed to be fully dynamic ($\alpha_{\text{height}} = 1$), accounting for the dynamic response of plant heights to environmental conditions. However, given that C_{leaf} is subject to the user defined adaptation rate α, the model considers some degree of conservatism in the growth form of the modelled plants between time steps, as large changes in height need to be accompanied by an adjustment of the leaf carbon pool. If a deciduous habit was evaluated as beneficial in the optimization function and $a_{\text{ll,new}}$ is below a threshold of 1 year after trait evolution, the modelled plant is considered deciduous.

Similarly, the climatic niche of the modelled plants are assumed to evolve towards local environmental conditions, subject to the adaptation rate α:

$$T_{\text{min,plant,new}} = T_{\text{min,plant,old}} + \mathcal{N}(\alpha, 0.05) \cdot (T_{\text{coldest month,local}} - T_{\text{min,plant,old}}) \tag{50}$$

$$T_{\text{max,plant,new}} = T_{\text{max,plant,old}} + \mathcal{N}(\alpha, 0.05) \cdot (T_{\text{warmest month,local}} - T_{\text{max,plant,old}}) \tag{51}$$

$$T_{\text{mean,plant,new}} = T_{\text{mean,plant,old}} + \mathcal{N}(\alpha, 0.05) \cdot (T_{\text{mean,local}} - T_{\text{mean,plant,old}}) \tag{52}$$

Besides trait adaptation, the model also considers changes in vegetation structure and functioning due to dispersal dynamics. At every time step, it is assumed that productive reference plants can disperse into surrounding grid cells within a user-defined range. If the migrating model plant with its specific trait combination can outcompete the already present model plant, it is assumed to occupy the location. Dispersal is modelled as a stochastic process, accounting for the various factors that can affect dispersal dynamics, but that are not explicitly represented in the model (e.g., wind and other environmental conditions, dispersal vectors) [34]. To do so, at every time step and for each modelled plant a new dispersal range is drawn from a right-skewed dispersal kernel, defined by a Weibull distribution with a shape parameter value of 1.75 and a user-defined scale parameter. The shape of the dispersal kernel accounts for the generally high frequency of short-distance dispersal and the low frequency of long-distance dispersal events [35]. In the current model, the scale parameter of the dispersal kernel is varied between 200 and 1100 km per 10 kyr, covering the dispersal distances inferred from fossil records for the Paleocene–Eocene Thermal Maximum [36].

If several reference plants with a positive NCG occur in a location after dispersal, a competition function selects the surviving reference plant with the best trait combination. Thereby, it is generally assumed that the reference plant with the highest NCG and plant height will dominate the local vegetation, considering carbon gain and light competition as the main fitness measures. To account for the combined height and NCG competition, the NCG is scaled with a height penalty function that depends on the average height of the competing reference plants [33].

For some locations, or after extreme environmental changes, it is possible that there is no reference plant that can maintain a positive carbon balance in the given environment. In this model step, it can be decided whether the reference plants occurring in these locations die out and are removed, or whether they are assumed to remain as unproductive occupants, imitating the possibility of local refugia (default assumption).

To model trait evolution, dispersal and competition dynamics, the described model functions are implemented in the “general engine for eco-evolutionary simulations” framework “gen3sis” [37]. The framework allows an efficient handling of the described eco-evolutionary steps while considering a spatially-explicit dynamic landscape with temporally variable climatic input data.

Supplementary Tables

Supplementary Table 1 TREED model parameters. Parameters for approximating vegetation structure, photosynthesis, respiration, carbon turnover and trait evolution.

Function	Parameter	Description	Value
Allometric relationships			
	$k_{la:sa}$	Leaf area to sapwood cross-section area	4000 [-]
	k_{allom2}	Height–diameter relationship	50 [-]
	k_{allom3}	Height–diameter relationship	0.6 [-]
	k_{allom1}	Diameter–crown area relationship	75 [-]
	k_{rp}	Diameter–crown area relationship	1.6 [-]
	DM_c	Average dry biomass carbon content	0.47 g C / g biomass
	WD	Average wood density	250000 g C / m^3
	$k_{intercept}$	Volume–biomass relationship	194 g biomass / m^3
	k_{pow}	Volume–biomass relationship	1.23 [-]
Carbon balance			
	$\alpha_{leaf:stand}$	Leaf to stand radiation use efficiency scaling	0.55 [-]
	α_{C3}	Intrinsic quantum efficiency for CO_2 uptake	0.08 [-]
	τ_{25}	Rubsico specifity factor at 25°C	2600 [-]
	Q_{10_τ}	Rubisco temperature sensitivity parameter	0.57 [-]
	K_C	Michaelis-Menten constant for CO_2	30 Pa
	K_O	Michaelis-Menten constant for O_2	30000 Pa
	$[O_2]$	Partial pressure of O_2 (default)	20900 Pa
	b	Leaf respiration coefficient	0.015 rate / day
	θ	Light and Rubisco co-limitation shape factor	0.7 [-]
	λ_{max}	Maximum leaf–atmosphere gas exchange	0.8 [-]
	g_{min}	Minimum canopy conductance	0.3 mm / s
	α_m	Pristley-Taylor coefficient	1.391 [-]
	g_m	Conductance scaling parameter	3.26 mm / s
	r	Base respiration rate	0.066 g C / g N / day
	$C{:}N_{wood}$	C to N ratio woody tissue	330 [-]
	$C{:}N_{root}$	C to N ratio root tissue	29 [-]
	r_{gr}	Growth respiration fraction	0.25 [-]
	$f_{sapwood}$	Sapwood carbon turnover time	0.05 / year
	$f_{heartwood}$	Heartwood carbon turnover time	0.05 / year
	$f_{coarseroot}$	Coarseroot carbon turnover time	0.05 / year
Trait dynamics			
	α_{trait}	Rate of trait adaptation per time step	0 to 1 [-]
	$k_{nichebreadth}$	Plant sensitivity to variation from climatic tolerance	0, 0.03 or 0.1 [-]

Supplementary Table 2 TREED model variables. Overview of model variables and their units.

Variable	Description	Unit
C_{leaf}	Average individual leaf carbon pool	g C / individual
$C_{coarseroot}$	Average individual coarseroot carbon pool	g C / individual
$C_{sapwood}$	Average individual sapwood carbon pool	g C / individual
$C_{heartwood}$	Average individual heartwood carbon pool	g C / individual
$C_{fineroot}$	Average individual fineroot carbon pool	g C / individual
H	Average individual plant height	m
phenology	deciduous or evergreen habit	[-]
r:s	root to shoot ratio	[-]
a_{ll}	average individual leaf longevity	years
LA	Average individual leaf area	m^2
SA	Average individual sapwood cross-section area	m^2
D	Average individual stem diameter	m
CA	Average individual crown area (occuped surface area)	m^2
SLA	Average individual specific leaf area	m^2 / g C
DM_c	Average dry biomass carbon content	g C / g dry biomass
$T_{max,local}$	Maximum monthly average temperature	°C
$T_{min,local}$	Minimum monthly average temperature	°C
$T_{mean,local}$	Annual mean temperature	°C
$T_{max,veg}$	Local vegetation's upper temperature niche limit	°C
$T_{min,veg}$	Local vegetation's lower temperature niche limit	°C
$T_{mean,veg}$	Local vegetation's optimum annual average temperature	°C
LAI	Average leaf area index at location	m^2 / m^2
FPC	Average foliage projective cover (light absorption potential)	[-]
PAR	Photosynthetically active radiation	MJ / m^2 /day
daylength	Length of day	hours
J_E	Rubisco-limited photosynthesis	mol C / m^2 / h
J_C	Light-limited photosynthesis	mol C / m^2 / h
p_a	Ambient partial pressure of CO_2	Pa
p_i	Leaf internal partial pressure of CO_2	Pa
Γ_*	Photorespiratory CO_2 compensation point	Pa
f_{temp}	Temperature limitation of photosynthesis	-
V_m	Maximum Rubisco capacity	mol C / m^2 / day
A_{gd}	Daily gross photosynthesis	g C / m^2 / daytime
A_{dt}	Net daytime photosynthesis	g C / m^2 / daytime
R_{leaf}	Leaf respiration	g C / m^2 / day
g_c	canopy conductance of water vapor	mm / s
E_{demand}	Vegetation water demand	mm / day
E_{eq}	Equilibrium evapotranspiration	mm / day
sa	slope of saturation vapor pressure curve	Pa / K
γ	psychrometric constant	J / kg
R_n	Daytime net radiation at surface	J / m^2 / day
λ_{vap}	Latent heat of vaporization	J / kg
AET	Actual evapotranspiration	mm/day
$R_{sapwood}$	Sapwood maintenance respiration	g C / m^2 / day
$R_{fineroot}$	Fineroot maintenance respiration	g C / m^2 / day
g(T)	Temperature-dependence of respiration	[-]
C_{soil}	Soil carbon content	g C / m^2
$\tau_{sapwood}$	Average sapwood carbon turnover	g C / m^2 / year
$\tau_{heartwood}$	Average heartwood carbon turnover	g C / m^2 / year
$\tau_{coarseroot}$	Average coarseroot carbon turnover	g C / m^2 / year
τ_{leaf}	Average leaf carbon turnover	g C / m^2 / year
$\tau_{fineroot}$	Average fineroot carbon turnover	g C / m^2 / year
HD	Heat stress induced mortality/turnover index	[-]
FD	Cold stress induced mortality/turnover index	[-]
NPP	Net primary productivity	g C / m^2 / year
NCG	Net carbon gain	g C / m^2 / year

Supplementary References

[1] Beck, H. E. *et al.* Present and future Köppen-Geiger climate classification maps at 1-km resolution. *Scientific Data* **5**, 180214 (2018). URL http://www.nature.com/articles/sdata2018214.

[2] Karger, D. N. *et al.* Climatologies at high resolution for the earth's land surface areas. *Scientific Data* **4**, 170122 (2017). URL https://www.nature.com/articles/sdata2017122.

[3] R Core Team. *R: A Language and Environment for Statistical Computing*. R Foundation for Statistical Computing, Vienna, Austria (2023). URL https://www.R-project.org/.

[4] Wickham, H. *ggplot2: Elegant Graphics for Data Analysis*. Springer-Verlag New York (2016). URL https://ggplot2.tidyverse.org

[5] Lang, N., Jetz, W., Schindler, K. & Wegner, J. D. A high-resolution canopy height model of the Earth. *Nature Ecology & Evolution* **7**, 1778–1789 (2023). URL https://www.nature.com/articles/s41559-023-02206-6.

[6] NASA/LARC/SD/ASDC. CERES Energy Balanced and Filled (EBAF) TOA and Surface Monthly means data in netCDF Edition 4.2 (2023). URL https://doi.org/10.5067/TERRA-AQUA-NOAA20/CERES/EBAF_L3B004.2.

[7] Kern, S. MODIS Collection 6.1 Sinusoidal Tiles Yearly gap-filled Gross and Net Primary Production (2024). URL http://doi.org/10.25592/uhhfdm.14633.

[8] Huang, Y. *et al.* A global map of root biomass across the world's forests. *Earth System Science Data* **13**, 4263–4274 (2021). URL https://essd.copernicus.org/articles/13/4263/2021/.

[9] Miralles, D. G. *et al.* Global land-surface evaporation estimated from satellite-based observations. *Hydrology and Earth System Sciences* **15**, 453–469 (2011). URL https://hess.copernicus.org/articles/15/453/2011/.

[10] Wang, H. *et al.* Leaf economics fundamentals explained by optimality principles. *Science Advances* **9**, eadd5667 (2023). URL https://www.science.org/doi/10.1126/sciadv.add5667.

[11] Wright, I. J. *et al.* The worldwide leaf economics spectrum. *Nature* **428**, 821–827 (2004). URL http://www.nature.com/articles/nature02403.

[12] Harper, K. *et al.* ESA Land Cover Climate Change Initiative (Land_cover_cci): Global Plant Functional Types (PFT) Dataset, v2.0.8. NERC EDS Centre for Environmental Data Analysis. (2023). URL https://dx.doi.org/10.5285/26a0f46c95ee4c29b5c650b129aab788.

[13] Hijmans, R. J. *raster: Geographic Data Analysis and Modeling.* R package version 3.6-26 (2023). URL https://CRAN.R-project.org/package=raster.

[14] Currano, E. D. *et al.* Sharply increased insect herbivory during the Paleocene–Eocene Thermal Maximum. *Proceedings of the National Academy of Sciences* **105**, 1960–1964 (2008). URL https://pnas.org/doi/full/10.1073/pnas.0708646105.

[15] Currano, E. D., Labandeira, C. C. & Wilf, P. Fossil insect folivory tracks paleotemperature for six million years. *Ecological Monographs* **80** (2010). URL https://esajournals.onlinelibrary.wiley.com/doi/epdf/10.1890/09-2138.1.

[16] Sitch, S. *et al.* Evaluation of ecosystem dynamics, plant geography and terrestrial carbon cycling in the LPJ dynamic global vegetation model. *Global Change Biology* **9**, 161–185 (2003). URL http://doi.wiley.com/10.1046/j.1365-2486.2003.00569.x.

[17] Schaphoff, S. *et al.* LPJmL4 – a dynamic global vegetation model with managed land – Part 1: Model description. *Geoscientific Model Development* **11**, 1343–1375 (2018). URL https://gmd.copernicus.org/articles/11/1343/2018/.

[18] Shinozaki, K., Yoda, K., Hozumi, K. & Kira, T. A quantitative analysis of plant form - the pipe model theory: I. Basic analyses. *Japanese Journal of Ecology* **14**, 97–105 (1964). URL https://doi.org/10.18960/seitai.14.3_97.

[19] Waring, R., Schroeder, P. & Oren, R. Application of pipe model theory to predict canopy leaf area. *Canadian Journal of Forest Research* **12**, 556–560 (1982). URL https://doi.org/10.1139/x82-086.

[20] Huang, S., Titus, S. J. & Wiens, D. P. Comparison of nonlinear height-diamter functions for major Alberta tree species. *Canadian Journal of Forest Research* **22**, 1297–1304 (1992). URL https://doi.org/10.1139/x92-172.

[21] Brunner, I. & Godbold, D. L. Tree roots in a changing world. *Journal of Forest Research* **12**, 78–82 (2007). URL https://www.tandfonline.com/doi/full/10.1007/s10310-006-0261-4.

[22] Farquhar, G. D., Von Caemmerer, S. & Berry, J. A. A biochemical model of photosynthetic CO2 assimilation in leaves of C3 species. *Planta* **149**, 78–90 (1980). URL http://link.springer.com/10.1007/BF00386231.

[23] Farquhar, G. D. & Caemmerer, S. in *Modelling of Photosynthetic Response to Environmental Conditions* (eds Lange, O. L., Nobel, P. S., Osmond, C. B. & Ziegler, H.) *Physiological Plant Ecology II: Water Relations and Carbon Assimilation* 549–587 (Springer, Berlin, 1982). OCLC: 840294129. URL https://doi.org/10.1007/978-3-642-68150-9_17.

[24] Collatz, G., Ball, J., Grivet, C. & Berry, J. A. Physiological and environmental regulation of stomatal conductance, photosynthesis and transpiration: a model that includes a laminar boundary layer. *Agricultural and Forest Meteorology* **54**, 107–136 (1991). URL https://linkinghub.elsevier.com/retrieve/pii/0168192391900028.

[25] Haxeltine, A. & Prentice, I. C. A General Model for the Light-Use Efficiency of Primary Production. *Functional Ecology* **10**, 551 (1996). URL https://www.jstor.org/stable/2390165?origin=crossref.

[26] Monteith, J. Accommodation between transpiring vegetation and the convective boundary layer. *Journal of Hydrology* **166**, 251–263 (1995). URL https://linkinghub.elsevier.com/retrieve/pii/002216949405086D.

[27] Reich, P. B. *et al.* Boreal and temperate trees show strong acclimation of respiration to warming. *Nature* **531**, 633–636 (2016). URL https://www.nature.com/articles/nature17142.

[28] Lloyd, J. & Taylor, J. A. On the temperature dependence of soil respiration. *Functional Ecology* **8**, 315–323 (1994). URL https://www.jstor.org/stable/2389824?origin=crossref.

[29] Todd-Brown, K. E. O. *et al.* Causes of variation in soil carbon simulations from CMIP5 Earth system models and comparison with observations. *Biogeosciences* **10**, 1717–1736 (2013). URL https://bg.copernicus.org/articles/10/1717/2013/.

[30] Franklin, O. *et al.* Organizing principles for vegetation dynamics. *Nature Plants* **6**, 444–453 (2020). URL https://www.nature.com/articles/s41477-020-0655-x.

[31] Harrison, S. P. *et al.* Eco-evolutionary optimality as a means to improve vegetation and land-surface models. *New Phytologist* **231**, 2125–2141 (2021). URL https://nph.onlinelibrary.wiley.com/doi/10.1111/nph.17558.

[32] Mullen, K., Ardia, D., Gil, D., Windover, D. & Cline, J. DEoptim: An *R* Package for Global Optimization by Differential Evolution. *Journal of Statistical Software* **40** (2011). URL http://www.jstatsoft.org/v40/i06/.

[33] Falster, D. S. & Westoby, M. Plant height and evolutionary games. *Trends in Ecology & Evolution* **18**, 337–343 (2003). URL https://linkinghub.elsevier.com/retrieve/pii/S0169534703000612.

[34] Lososová, Z. *et al.* Seed dispersal distance classes and dispersal modes for the European flora. *Global Ecology and Biogeography* **32**, 1485–1494 (2023). URL https://onlinelibrary.wiley.com/doi/10.1111/geb.13712.

[35] Corlett, R. T. & Westcott, D. A. Will plant movements keep up with climate change? *Trends in Ecology & Evolution* **28**, 482–488 (2013). URL https://linkinghub.elsevier.com/retrieve/pii/S0169534713001055.

[36] Wing, S. L. *et al.* Transient Floral Change and Rapid Global Warming at the Paleocene-Eocene Boundary. *Science* **310**, 993–996 (2005). URL https://www.science.org/doi/10.1126/science.1116913.

[37] Hagen, O. *et al.* gen3sis: A general engine for eco-evolutionary simulations of the processes that shape Earth's biodiversity. *PLOS Biology* **19**, e3001340 (2021). URL https://dx.plos.org/10.1371/journal.pbio.3001340.

Full reference list of TRY trait data contributions considered in this study

Abakumova, M., Zobel, K., Lepik, A., Semchenko, M. et al. Plasticity in plant functional traits is shaped by variability in neighbourhood species composition. New Phytol. 211, 455-463 (2016).

Adler, P. B. et al. Functional traits explain variation in plant life history strategies. Proc. Natl Acad. Sci. U.S.A. 111, 740-745 (2014).

Adler, P. B., Milchunas, D. G., Lauenroth, W. K., Sala, O. E. & Burke, I. C. Functional traits of graminoids in semi-arid steppes: a test of grazing histories. J. Appl. Ecol. 41, 653-663 (2004).

Ali, H. E., Reineking, B. & Münkemüller, T. Plant Soil 411, 359 (2017).

Anderson, C. G., Bond-Lamberty, B. & Stegen, J. C. Controls on and consequences of specific leaf area variation with permafrost depth in a boreal forest. Submitted.

Apgaua, D. M., Ishida, F. Y., Tng, D. Y., Laidlaw, M. J., Santos, R. M. et al. Functional traits and water transport strategies in lowland tropical rainforest trees. PLoS ONE 10, e0130799 (2015).

Aspinwall, M. J., Pfautsch, S., Tjoelker, M. G., Varhammar, A., Possell, M. et al. Range size and growth temperature influence Eucalyptus species responses to an experimental heatwave. Glob. Change Biol. 25, 1665-1684 (2019).

Atkin, O. K. et al. Global variability in leaf respiration among plant functional types in relation to climate and leaf traits. New Phytol. 206, 614-636 (2015).

Atkin, O. K., Schortemeyer, M., McFarlane, N. & Evans, J. R. The response of fast- and slow-growing Acacia species to elevated atmospheric CO2: an analysis of the underlying components of relative growth rate. Oecologia 120, 544-554 (1999).

Atkin, O. K., Westbeek, M. H. M., Cambridge, M. L., Lambers, H. & Pons, T. L. Leaf respiration in light and darkness - a comparison of slow- and fast-growing Poa species. Plant Physiol. 113, 961-965 (1997).

Aubin, I. & Ricard, J. P. Ensemencement d'espèces compatibles dans les emprises de distribution d'électricité. Hydro-Québec, Unité Environnement – Direction Projets de distribution, 70 pp. (2000).

Aubin, I., Messier, C., Gachet, S., Lawrence, K., McKenney, D. et al. TOPIC—traits of plants in Canada. Natural Resources Canada, Canadian Forest Service, Sault Ste. Marie, Ontario (2012).

Auger, S. & Shipley, B. Interspecific and intraspecific trait variation along short environmental gradients in an old-growth temperate forest. J. Veg. Sci. (2012).

Baastrup-Spohr, L., Sand-Jensen, K., Nicolajsen, S. V. & Bruun, H. H. From soaking wet to bone dry: predicting plant community composition along a steep hydrological gradient. J. Veg. Sci. 26, 619–630 (2015).

Bahar, N. H. A. et al. Leaf-level photosynthetic capacity in lowland Amazonian and high-elevation Andean tropical moist forests of Peru. New Phytol. 214, 1002-1018 (2017).

Bahn, M., Wohlfahrt, G., Haubner, E., Horak, I., Michaeler, W. et al. Leaf photosynthesis, nitrogen contents and specific leaf area of 30 grassland species in differently managed mountain ecosystems in the Eastern Alps. In Cernusca, A., Tappeiner, U. & Bayfield, N. (eds.) Land-use changes in European mountain ecosystems. ECOMONT - Concept and Results, 247-255 (Blackwell Wissenschaft, Berlin, 1999).

Balazs, K., Kramer, A., Munson, S. & Butterfield, B. The right trait in the right place at the right time: matching traits to environment improves restoration outcomes. Ecol. Appl. 30, e02022 (2020).

Baraloto, C., Paine, C. E. T., Poorter, L., Beauchene, J., Bonal, D. et al. Decoupled leaf and stem economics in rainforest trees. Ecol. Lett. 13, 1338-1347 (2010).

Baruch, Z. & Goldstein, G. Leaf construction cost, nutrient concentration, and net CO2 assimilation of native and invasive species in Hawaii. Oecologia 121, 183-192 (1999).

Baruch, Z., Christmas, M., Breed, M. F., Guerin, G. R., Caddy-Retalic, S. et al. Leaf trait associations with environmental variation in the wide-ranging shrub Dodonaea viscosa subsp. angustissima (Sapindaceae). Austral Ecol. 43, 553-561 (2017).

Beckmann, M., Hock, M., Bruelheide, H. & Erfmeier, A. The role of UV-B radiation in the invasion of Hieracium pilosella—A comparison of German and New Zealand plants. Environ. Exp. Bot. 75, 173–180 (2012).

Belluau, M. & Shipley, B. Linking hard and soft traits: physiology, morphology and anatomy interact to determine habitat affinities to soil water availability in herbaceous dicots. PLoS ONE 13, e0193130 (2018).

Berner, L. T., Alexander, H. D., Loranty, M. M., Ganzlin, P., Mack, M. C. et al. Biomass allometry for alder, dwarf birch, and willow in boreal forest and tundra ecosystems of far northeastern Siberia and north-central Alaska. For. Ecol. Manage. 337, 110-118 (2015).

Bjorkman, A. D., Myers-Smith, I. H., Elmendorf, S. C., Normand, S., Thomas, H. J. D. et al. Tundra Trait Team: a database of plant traits spanning the tundra biome. Glob. Ecol. Biogeogr. 27, 1402-1411 (2018).

Blonder, B. et al. The shrinkage effect biases estimates of paleoclimate. Am. J. Bot. 99, 1756-1763 (2012).

Blonder, B., Baldwin, B., Enquist, B. J. & Robichaux, R. H. Variation and macroevolution in leaf functional traits in the Hawaiian silversword alliance (Asteraceae). J. Ecol. 104, 219–228 (2016).

Blonder, B., Kapas, R. E., Dalton, R. M., Graae, B. J., Heiling, J. M. et al. Microenvironment and functional-trait context dependence predict alpine plant community dynamics. J. Ecol. 106, 1323-1337 (2018).

Blonder, B., Violle, C. & Enquist, B. J. Assessing the causes and scales of the leaf economics spectrum using venation networks in Populus tremuloides. J. Ecol. 101, 981–989 (2013).

Blonder, B., Violle, C., Patrick, L. & Enquist, B. Leaf venation networks and the origin of the leaf economics spectrum. Ecol. Lett. 14, 91-100 (2011).

Bond-Lamberty, B., Wang, C. & Gower, S. T. Above- and belowground biomass and sapwood area allometric equations for six boreal tree species of northern Manitoba. Can. J. For. Res. 32, 1441-1450 (2002).

Bond-Lamberty, B., Wang, C. & Gower, S. T. Leaf area dynamics of a boreal black spruce fire chronosequence. Tree Physiol. 22, 993-1001 (2002).

Boukili, V. K. & Chazdon, R. L. Environmental filtering, local site factors and landscape context drive changes in functional trait composition during tropical forest succession. Perspect. Plant Ecol. Evol. Syst. 24, 37-47 (2017).

Bragazza, L. Conservation priority of Italian alpine habitats: a floristic approach based on potential distribution of vascular plant species. Biodivers. Conserv. 18, 2823-2835 (2009).

Brendel, M. R., Schurr, F. M. & Sheppard, C. S. Inter- and intraspecific selection in alien plants: how population growth, functional traits and climate responses change with residence time. Glob. Ecol. Biogeogr. (2021).

Bricca, A., Conti, L., Tardella, M. F., Catorci, A., Iocchi, M. et al. Community assembly processes along a sub-Mediterranean elevation gradient: analyzing the interdependence of trait community weighted mean and functional diversity. Plant Ecol. 220, 1139-1151 (2019).

Brown, K. A. et al. Use of provisioning ecosystem services drives loss of functional traits across land use intensification gradients in tropical forests in Madagascar. Biol. Conserv. 161, 118-127 (2013).

Brumnich, F., Marchetti, Z. Y. & Pereira, M. S. Changes in forest diversity over a chronosequence of fluvial islands. iForest 12, 306-316 (2019).

Bucher, S. F., Auerswald, K., Tautenhahn, S., Geiger, A., Otto, J. et al. Intra- and interspecific variation in stomatal pore area index along altitudinal gradients and its relation to leaf functional traits. Plant Ecol. 217, 229-240 (2016).

Burrascano, S., Copiz, R., Del Vico, E., Fagiani, S., Giarrizzo, E. et al. Wild boar rooting intensity determines shifts in understorey composition and functional traits. Community Ecol. 16, 244-253 (2015).

Butterfield, B. J. & Briggs, J. M. Regeneration niche differentiates functional strategies of desert woody plant species. Oecologia 165, 477-487 (2011).

Byun, C., de Blois, S. & Brisson, J. Plant functional group identity and diversity determine biotic resistance to invasion by an exotic grass. J. Ecol. (2013).

Cadotte, M. W. Functional traits explain ecosystem function through opposing mechanisms. Ecology Letters 20, 989-996 (2017).

Campany, C. E., Martin, L. & Watkins, J. E. Convergence of ecophysiological traits drives floristic composition of early lineage vascular plants in a tropical forest floor. Ann. Bot. 121, 1-12 (2018).

Campbell, C., Atkinson, L., Zaragoza-Castells, J., Lundmark, M., Atkin, O. et al. Acclimation of photosynthesis and respiration is asynchronous in response to changes in temperature regardless of plant functional group. New Phytol. 176, 375-389 (2007).

Campetella, G., Botta-Dukát, Z., Wellstein, C., Canullo, R., Gatto, S. et al. Patterns of plant trait-environment relationships along a forest succession chronosequence. Agric. Ecosyst. Environ. 145, 38-48 (2011).

Campetella, G., Chelli, S., Wellstein, C., Farris, E., Calvia, G. et al. Contrasting patterns in leaf traits of Mediterranean shrub communities along an elevation gradient: measurements matter. Plant Ecol. (2019).

Carswell, F. E., Meir, P., Wandelli, E. V., Bonates, L. C. M., Kruijt, B. et al. Photosynthetic capacity in a central Amazonian rain forest. Tree Physiol. 20, 179-186 (2000).

Carvalho, F., Brown, K. A., Waller, M. P., Bunting, M. J., Boom, A. et al. A method for reconstructing temporal changes in vegetation functional trait composition using Holocene pollen assemblages. PLoS ONE 14, e0216698 (2019).

Castro-Diez, P., Puyravaud, J. P., Cornelissen, J. H. C. & Villar-Salvador, P. Stem anatomy and relative growth rate in seedlings of a wide range of woody plant species and types. Oecologia 116, 57-66 (1998).

Catford, J. A., Morris, W. K., Vesk, P. A., Gippel, C. J. & Downes, B. J. Species and environmental characteristics point to flow regulation and drought as drivers of riparian plant invasion. Divers. Distrib. 20, 1084-1096 (2014).

Cavender-Bares, J., Keen, A. & Miles, B. Phylogenetic structure of floridian plant communities depends on taxonomic and spatial scale. Ecology 87, S109-S122 (2006).

Cavender-Bares, J., Sack, L. & Savage, J. Atmospheric and soil drought reduce nocturnal conductance in live oaks. Tree Physiol. 27, 611-620 (2007).

Cerabolini, B. E. L., Brusa, G., Ceriani, R. M., De Andreis, R., Luzzaro, A. et al. Can CSR classification be generally applied outside Britain? Plant Ecol. 210, 253-261 (2010).

Cerabolini, B., Pierce, S., Luzzaro, A. & Ossola, A. Species evenness affects ecosystem processes in situ via diversity in the adaptive strategies of dominant species. Plant Ecol. 207, 333-345 (2010).

Chacón-Madrigal, E., Wanek, W., Hietz, P. & Dullinger, S. Traits indicating a conservative resource strategy are weakly related to narrow range size in a group of neotropical trees. Perspect. Plant Ecol. Evol. Syst. (2018).

Chen, et al. Species-independent down-regulation of leaf photosynthesis and respiration in response to shading: evidence from six temperate tree species. PLoS ONE 9, e91798 (2014).

Chen, Y., Han, W., Tang, L., Tang, Z. & Fang, J. Leaf nitrogen and phosphorus concentrations of woody plants differ in responses to climate, soil and plant growth form. Ecography 34, 76-84 (2011).

Choat, B., Jansen, S., Brodribb, T. S., Cochard, H., Delzon, S. et al. Global convergence in the vulnerability of forests to drought. Nature 491, 752-755 (2012).

Ciccarelli, D. Mediterranean coastal dune vegetation: are disturbance and stress the key selective forces that drive the psammophilous succession? Estuar. Coast. Shelf Sci. 165, 247-253 (2015).

Ciocarlan, V. The illustrated Flora of Romania. Pteridophyta et Spermatopyta (Editura Ceres, 2009).

Coomes, D. A., Heathcote, S., Godfrey, E. R., Shepherd, J. J. & Sack, L. Scaling of xylem vessels and veins within the leaves of oak species. Biol. Lett. 4, 302-306 (2008).

Cornelissen, J. H. C. An experimental comparison of leaf decomposition rates in a wide range of temperate plant species and types. J. Ecol. 84, 573-582 (1996).

Cornelissen, J. H. C. et al. Functional traits of woody plants: correspondence of species rankings between field adults and laboratory-grown seedlings? J. Veg. Sci. 14, 311-322 (2003).

Cornelissen, J. H. C., Diez, P. C. & Hunt, R. Seedling growth, allocation and leaf attributes in a wide range of woody plant species and types. J. Ecol. 84, 755-765 (1996).

Cornelissen, J. H. C., Quested, H. M., Gwynn-Jones, D., Van Logtestijn, R. S. P., De Beus et al. Leaf digestibility and litter decomposability are related in a wide range of subarctic plant species and types. Funct. Ecol. 18, 779-786 (2004).

Cornwell, W. K. et al. Plant species traits are the predominant control on litter decomposition rates within biomes worldwide. Ecol. Lett. 11, 1065-1071 (2008).

Cornwell, W. K., Bhaskar, R., Sack, L., Cordell, S. & Lunch, C. K. Adjustment of structure and function of Hawaiian Metrosideros polymorpha at high vs. low precipitation. Funct. Ecol. 21, 1063-1071 (2007).

Craine, J. M., Nippert, J. B., Towne, E. G., Tucker, S., Kembel, S. W. et al. Functional consequences of climate-change induced plant species loss in a tallgrass prairie. Oecologia 165, 1109-1117 (2011).

Craven, D., Braden, D., Ashton, M. S., Berlyn, G. P., Wishnie, M. et al. Between- and within-site comparisons of structural and physiological characteristics and foliar nutrient content of 14 tree species at a wet, fertile site and a dry, infertile site in Panama. For. Ecol. Manage. 238, 335-346 (2007).

Cunliffe, A. M., Assmann, J., Daskalova, G., Kerby, J. T. & Myers-Smith, I. H. Aboveground biomass corresponds strongly with drone-derived canopy height but weakly with greenness (NDVI) in a shrub tundra landscape. Environ. Res. Lett. (2020).

Cunliffe, A. M., McIntire, C. D., Boschetti, F., Sauer, K. J., Litvak, M. et al. Allometric relationships for predicting aboveground biomass and sapwood area of Oneseed Juniper (Juniperus monosperma) trees. Front. Plant Sci. 11, 94 (2021).

Cunliffe, A., Anderson, K., Boschetti, F., Brazier, R., Graham, H. et al. Global application of a drone photogrammetry protocol for predicting aboveground biomass in non-forest ecosystems. Preprint on bioRxiv (In Review).

Dahlin, K. M., Asner, G. P. & Field, C. B. Environmental and community controls on plant canopy chemistry in a Mediterranean-type ecosystem. Proc. Natl Acad. Sci. U.S.A. 110, 6895-6900 (2013).

Dalke, I. V., Novakovskiy, A. B., Maslova, S. P. et al. Plant Ecol. 219, 1295 (2018).

Dang-Le Anh Tuan. Characterization growth of Rhizophora apiculata Blume at Can Gio Mangrove Biosphere Reserve, Vietnam. Unpublished data.

Dawson, S. K., Warton, D. I., Kingsford, R. T., Berney, P., Keith, D. A. et al. Plant traits of propagule banks and standing vegetation reveal flooding alleviates impacts of agriculture on wetland restoration. J. Appl. Ecol. 54, 1907-1918 (2017).

De Frutos, A., Navarro, T., Pueyo, Y. & Alados, C. L. Inferring resilience to fragmentation-induced changes in plant communities in a semi-arid Mediterranean ecosystem. PLoS ONE 10, e0118837 (2015).

De Frutos, Á., Navarro, T., Pueyo, Y. & Alados, C. L. Inferring resilience to fragmentation-induced changes in plant communities in a semi-arid Mediterranean ecosystem. PLoS ONE 10, e0118837 (2015).

De Long, J. R., Jackson, B. G., Wilkinson, A. et al. Relationships between plant traits, soil properties and carbon fluxes differ between monocultures and mixed communities in temperate grassland. J. Ecol. 107, 1704–1719 (2019).

De Vries, F. & Bardgett, R. D. Plant community controls on short-term ecosystem nitrogen retention. New Phytol. 210, 1049-1059 (2016).

Dechant, B., Cuntz, M., Vohland, M., Schulz, E. & Doktor, D. Estimation of photosynthesis traits from leaf reflectance spectra: correlation to nitrogen content as the dominant mechanism. Remote Sens. Environ. 196, 279-292 (2017).

Delpierre, N., Berveiller, D., Granda, E. & Dufrêne, E. Wood phenology, not carbon input, controls the interannual variability of wood growth in a temperate oak forest. New Phytol. 210, 459-470 (2016).

Díaz, S., Hodgson, J. G., Thompson, K., Cabido, M., Cornelissen, J. H. C. et al. The plant traits that drive ecosystems: evidence from three continents. J. Veg. Sci. 15, 295-304 (2004).

Domingues, T. F., Martinelli, L. A. & Ehleringer, J. R. Ecophysiological traits of plant functional groups in forest and pasture ecosystems from eastern Amazonia, Brazil. Plant Ecol. 193, 101–112 (2007).

Domingues, T. F., Meir, P., Feldpausch, T. R. et al. Co-limitation of photosynthetic capacity by nitrogen and phosphorus in West Africa woodlands. Plant Cell Environ. 33, 959-980 (2010).

Dong, N., Prentice, I. C., Evans, B. J., Caddy-Retalic, S., Lowe, A. J. et al. Leaf nitrogen from first principles: field evidence for adaptive variation with climate. Biogeosciences 14, 481-495 (2017).

Dong, N., Prentice, I. C., Wright, I. J., Evans, B. J., Togashi, H. F. et al. Components of leaf-trait variation along environmental gradients. New Phytol., (in press).

Douma, J. C., Bardin, V., Bartholomeus, R. P. & van Bodegom, P. M. Quantifying the functional responses of vegetation to drought and oxygen stress in temperate ecosystems. Funct. Ecol. 26, 1355-1365 (2012).

Dwyer, J. M., Hobbs, R. J. & Mayfield, M. M. Specific leaf area responses to environmental gradients through space and time. Ecology 95, 399-410 (2014).

Everwand, G., Fry, E. L., Eggers, T. & Manning, P. Seasonal variation in the relationship between plant traits and grassland carbon and water fluxes. Ecosystems 17, 1095-1108 (2014).

E-Vojtkó, A. et al. Leaf trait records of vascular plant species in the Pannonian flora with special focus on endemics and rarities. Folia Geobot. 55, 73-79 (2020).

Falster, D. S., Duursma, R. A., Ishihara, M. I., Barneche, D. R., FitzJohn, R. G. et al. BAAD: a biomass and allometry database for woody plants. Ecology 96, 1445 (2015).

Fan, Y., Miguez-Macho, G., Jobbágy, E. G., Jackson, R. B. & Otero-Casal, C. Hydrologic regulation of plant rooting depth. Proc. Natl Acad. Sci. U.S.A. 114, 10572-10577 (2017).

Fazlioglu, F., Wan, J. S. H. & Bonser, S. P. Phenotypic plasticity and specialization along an altitudinal gradient in Trifolium repens. Turk. J. Bot. 42, 440–447 (2018).

Feng, Y. & van Kleunen, M. Responses to shading of naturalized and non-naturalized exotic woody species. Ann. Bot. 114, 981-989 (2014).

Findurova, A. Variability of leaf traits SLA and LDMC in selected species of the Czech flora. Ms thesis, Masaryk University, Brno (2018).

Finegan, B. et al. Does functional trait diversity predict above-ground biomass and productivity of tropical forests? J. Ecol. 103, 191-201 (2015).

Fitter, A. H. & Peat, H. J. The Ecological Flora Database. J. Ecol. 82, 415-425 (1994).

Fonseca, C. R., Overton, J. M., Collins, B. & Westoby, M. Shifts in trait-combinations along rainfall and phosphorus gradients. J. Ecol. 88, 964-977 (2000).

Forgiarini, C., Souza, A. F., Longhi, S. J. & Oliveira, J. M. In the lack of extreme pioneers: trait relationships and ecological strategies of 66 subtropical tree species. J. Plant Ecol. 8, 359-367 (2015).

Fortunel, C., McFadden, I. R., Valencia, R. & Kraft, N. J. B. Neither species geographic range size, climatic envelope nor intraspecific leaf trait variability capture habitat specialization in a hyperdiverse Amazonian forest. Biotropica (2019).

Frenette-Dussault, C., Shipley, B., Léger, J. F., Meziane, D. & Hingrat, Y. Functional structure of an arid steppe plant community reveals similarities with Grime's C-S-R theory. J. Veg. Sci. 23, 208-222 (2012).

Freschet, G. T., Cornelissen, J. H. C., van Logtestijn, R. S. P. & Aerts, R. Evidence of the 'plant economics spectrum' in a subarctic flora. J. Ecol. 98, 362-373 (2010).

Freschet, G. T., Kichenin, E. & Wardle, D. A. Explaining within-community variation in plant biomass allocation: a balance between organ biomass and morphology above vs below ground? J. Veg. Sci. 26, 431-440 (2015).

Freschet, G. T., Swart, E. M. & Cornelissen, J. H. C. Integrated plant phenotypic responses to contrasting above- and below-ground resources: key roles of specific leaf area and root mass fraction. New Phytol. 206, 1247-1260 (2015).

Freschet, G. T., Violle, C., Bourget, M. Y., Scherer-Lorenzen, M. & Fort, F. Allocation, morphology, physiology, architecture: the multiple facets of plant above- and belowground responses to resource stress. New Phytol. 219, 1338-1352 (2018).

Fry, E. L., Power, S. A. & Manning, P. Trait based classification and manipulation of functional groups in biodiversity-ecosystem function experiments. J. Veg. Sci. 25, 248-261 (2014).

Fyllas, N. M., Patino, S., Baker, T. R., Bielefeld Nardoto, G. et al. Basin-wide variations in foliar properties of Amazonian forest: phylogeny, soils and climate. Biogeosciences 6, 2677-2708 (2009).

Gachet, S., Vela, E. & Tatoni, T. BASECO: a floristic and ecological database of Mediterranean French flora. Biodivers. Conserv. 14, 1023-1034 (2005).

Garnier, E., Lavorel, S., Ansquer, P., Castro, H., Cruz, P. et al. Assessing the effects of land-use change on plant traits, communities and ecosystem functioning in grasslands: a standardized methodology and lessons from an application to 11 European sites. Ann. Bot. 99, 967-985 (2007).

Gentili, R., Gilardelli, F., Ciappetta, S., Ghiani, A. & Citterio, S. Inducing competition: intensive grassland seeding to control Ambrosia artemisiifolia. Weed Res. 55, 278-288 (2015).

Giarrizzo, E., Burrascano, S., Chiti, T., de Bello, F., Leps, J. et al. Re-visiting historical semi-natural grasslands in the Apennines to assess patterns of changes in plant species composition and functional traits. Appl. Veg. Sci. 20, 247-258 (2017).

Gonzalez-Akre, E., McShea, W., Bourg, N. & Anderson-Teixeira, K. Leaf traits data (SLA) for 56 woody species at the Smithsonian Conservation Biology Institute-ForestGEO Forest Dynamic Plot. Front Royal, VA, USA. Version 1.0 (2015).

Gos, P., Loucougaray, G., Colace, M. P., Arnoldi, C., Gaucherand, S. et al. Oecologia 180, 1001 (2016).

Green, W. USDA PLANTS Compilation, version 1, 09-02-02. NRCS: The PLANTS Database, National Plant Data Center, Baton Rouge, LA (http://plants.usda.gov) (2009).

Gubsch, M., Buchmann, N., Schmid, B., Schulze, E.-D., Lipowsky, A. et al. Differential effects of plant diversity on functional trait variation of grass species. Ann. Bot. 107, 157-169 (2011).

Gutiérrez, A. G. & Huth, A. Successional stages of primary temperate rainforests of Chiloé Island, Chile. Perspect. Plant Ecol. Syst. Evol. 14, 243-256 (2012).

Guy, A. L., Mischkolz, J. M. & Lamb, E. G. Limited effects of simulated acidic deposition on seedling survivorship and root morphology of endemic plant taxa of the Athabasca Sand Dunes in well-watered greenhouse trials. Botany 91, 176-181 (2013).

Han, W. X., Fang, J. Y., Guo, D. L. & Zhang, Y. Leaf nitrogen and phosphorus stoichiometry across 753 terrestrial plant species in China. New Phytol. 168, 377-385 (2005).

Han, W., Chen, Y., Zhao, F.-J., Tang, L., Jiang, R. et al. Floral, climatic and soil pH controls on leaf ash content in China's terrestrial plants. Glob. Ecol. Biogeogr. 21, 1001-1012 (2012).

Hao, G. Y., Sack, L., Wang, A. Y., Cao, K. F. & Goldstein, G. Differentiation of leaf water flux and drought tolerance traits in hemiepiphytic and non-hemiepiphytic Ficus tree species. Funct. Ecol. 24, 731-740 (2010).

Hattermann, D., Elstner, C., Bernhardt-Römermann, M. & Eckstein, L. Measurements from the project "Relative effects of local and regional factors as drivers for plant community diversity, functional trait diversity and genetic structure of species on Baltic uplift islands." (DFG: BE 4143/5-1 and EC 209/12-1).

Hayes, F. J., Buchanan, S. W., Coleman, B., Gordon, A. M., Reich, P. B. et al. Intraspecific variation in soy across the leaf economics spectrum. Ann. Bot. 123, 107-120 (2018).

He, P. et al. Leaf mechanical strength and photosynthetic capacity vary independently across 57 subtropical forest species with contrasting light requirements. New Phytol. 223, 607-618 (2019).

He, T., Lamont, B. B. & Downs, K. S. Banksias born to burn. New Phytol. 191, 184-196 (2011).

Heberling, J. M. & Mason, N. W. H. Are endemics functionally distinct?: Leaf traits of native and invasive woody species in a New Zealand forest. PLoS ONE 13, e0196746 (2019).

Heberling, J. M., Cassidy, S. T., Fridley, J. D. & Kalisz, S. Carbon gain phenologies of spring-flowering perennials in a deciduous forest indicate a novel niche for a widespread invader. New Phytol. 221, 778-788 (2019).

Helsen, K., Matsushima, H., Somers, B. & Honnay, O. A trait-based approach across the native and invaded range to understand plant invasiveness and community impact. Oikos (in press).

Helsen, K., Smith, S. W., Brunet, J. et al. Impact of an invasive alien plant on litter decomposition along a latitudinal gradient. Ecosphere 9, e02097 (2018).

Helsen, K., Van Cleemput, E., Bassi, L., Graae, B. J., Somers, B. et al. Inter- and intraspecific trait variation shape multidimensional trait overlap between two plant invaders and the invaded communities. Oikos 129, 677-688 (2020).

Herz, K., Dietz, S., Haider, S., Jandt, U., Scheel, D. et al. Drivers of intraspecific trait variation of grass and forb species in German meadows and pastures. J. Veg. Sci. 28, 705-716 (2017).

Herz, K., Dietz, S., Haider, S., Jandt, U., Scheel, D. et al. Predicting individual plant performance in grasslands. Ecol. Evol. 7, 8958-8965 (2017).

Hickler, T. Plant functional types and community characteristics along environmental gradients on Öland's Great Alvar (Sweden). Masters Thesis, University of Lund, Sweden (1999).

Higuchi, P. & Silva, A. C. Araucaria Forest Database (2013).

Hill, M. O., Preston, C. D. & Roy, D. B. PLANTATT - attributes of British and Irish Plants: status, size, life history, geography and habitats (Centre for Ecology and Hydrology, Huntingdon, 2004).

Hipp, A. L., Glasenhardt, M.-C., Bowles, M. L., Garner, M., Scharenbroch, B. C. et al. Effects of phylogenetic diversity and phylogenetic identity in a restoration ecology experiment. In Scherson, R. A. & Faith, D. P. (eds.) Phylogenetic Diversity: Applications and Challenges in Biodiversity Science, 189-210 (Springer, Cham, 2018).

Hogan, J. A., Valverde-Barrantes, O. J., Xu, H. & Baraloto, C. Intraspecific root and leaf trait variation with tropical forest successional status: consequences for community-weighted patterns. (in review).

Hoof, J., Sack, L., Webb, D. T. & Nilsen, E. T. Contrasting structure and function of pubescent and glabrous varieties of Hawaiian Metrosideros polymorpha (Myrtaceae) at high elevation. Biotropica 40, 113-118 (2008).

Hough-Snee, N., Nackley, L. L., Kim, S. & Ewing, K. Does plant performance under stress explain divergent life history strategies? The effects of flooding and nutrient stress on two wetland sedges. Aquat. Bot. 120B, 151–159 (2015).

Iversen, C. M. et al. A global Fine-Root Ecology Database to address below-ground challenges in plant ecology. New Phytol. 215, 15-26 (2017).

Jactel, H. & Castagneyrol, B. Effect of drought on maritime pine needle traits in ORPHEE. (2019).

Jager, M. et al. Journal of Ecology 103, 374-385 (2015); Simpson, K. et al. Glob. Ecol. Biogeogr. 25, 964-978 (2016).

Jentsch, A. Disturbance Ecology, University of Bayreuth. Sampled by P. Aurich, K. Krapf, T.-Y. Chiu.

Joseph, G. S., Seymour, C. L., Cumming, G. S., Cumming, D. H. M. & Mahlangu, Z. Termite mounds increase functional diversity of woody plants in African savannas. Ecosystems 17, 808-819 (2014).

Junker, R. R., Lechleitner, M. H., Kuppler, J. et al. Interconnectedness of the Grinnellian and Eltonian niche in regional and local plant-pollinator communities. Front. Plant Sci. (in revision).

Kabzems, R. EP 1133 Aspen foliar Sierra / Fort Nelson. BC Ministry of Forests, Lands and Natural Resource Operations. Unpublished data.

Kaplan, Z., Danihelka, J., Chrtek, J. jun., Kirschner, J., Kubát, K. et al. Klíč ke květeně České republiky [Key to the flora of the Czech Republic], 2nd ed. (Academia, Praha, 2019).

Karbstein, K., Prinz, K., Hellwig, F. & Römermann, C. Plant intraspecific functional trait variation is related to within-habitat heterogeneity and genetic diversity in Trifolium montanum L. Ecol. Evol. 10, 5015–5033 (2020).

Kattenborn, T. & Schmidtlein, S. Radiative transfer modelling reveals why canopy reflectance follows function. Sci. Rep. 9, 6541 (2019).

Kattenborn, T., Fassnacht, F. E. & Schmidtlein, S. Differentiating plant functional types using reflectance: which traits make the difference? Remote Sens. Ecol. Conserv. 1-15 (2018).

Kattge, J., Knorr, W., Raddatz, T. & Wirth, C. Quantifying photosynthetic capacity and its relationship to leaf nitrogen content for global-scale terrestrial biosphere models. Glob. Change Biol. 15, 976-991 (2009).

Kearsley, E., Verbeeck, H., Hufkens, K., Van de Perre, F., Doetterl, S. et al. Functional community structure of African monodominant Gilbertiodendron dewevrei forest influenced by local environmental filtering. Ecol. Evol. 7, 295-304 (2017).

Kempel, A., Chrobock, T., Fischer, M., Rohr, R. P. & van Kleunen, M. Determinants of plant establishment success in a multispecies introduction experiment with native and alien species. Proc. Natl Acad. Sci. USA 110, 12727-12732 (2013).

Khalil, M. I., Gibson, D. J., Baer, S. G. & Willand, J. E. Functional diversity is more sensitive to biotic filters than phylogenetic diversity during community assembly. Ecosphere 9, e02164 (2018).

Kichenin, et al. Contrasting effects of plant inter- and intraspecific variation on community-level trait measures along an environmental gradient. Funct. Ecol. (2013).

Kirkup, D., Malcolm, P., Christian, G. & Paton, A. Towards a digital African Flora. Taxon 54, 457-466 (2005).

Kissling, D. W., Balslev, H., Baker, W. J., Dransfield, J., Göldel, B. et al. PalmTraits 1.0, a species-level functional trait database of palms worldwide. Sci. Data (2019).

Kleyer, M., Bekker, R. M., Knevel, I. C., Bakker, J. P., Thompson, K. et al. The LEDA Traitbase: a database of life-history traits of the Northwest European flora. J. Ecol. 96, 1266-1274 (2008).

Knauer, J. et al. Towards physiologically meaningful water-use efficiency estimates from eddy covariance data. Glob. Change Biol. 23, 2400-2418 (2017).

Koike, F. Plant traits as predictors of woody species dominance in climax forest communities. J. Veg. Sci. 12, 327-336 (2001).

Komac, B., Pladevall, C., Domenech, M. & Fanlo, R. Functional diversity and grazing intensity in sub-alpine and alpine grasslands in Andorra. Appl. Veg. Sci. (2014).

Kraft, N. J. B., Valencia, R. & Ackerly, D. Functional traits and niche-based tree community assembly in an Amazonian forest. Science 322, 580-582 (2008).

Kumarathunge, D. P. et al. Acclimation and adaptation components of the temperature dependence of plant photosynthesis at the global scale. New Phytol. 222, 768-784 (2019).

Kurokawa, H. & Nakashizuka, T. Leaf herbivory and decomposability in a Malaysian tropical rain forest. Ecology 89, 2645-2656 (2008).

La Pierre, K. J. & Smith, M. D. Functional trait expression of grassland species shift with short- and long-term nutrient additions. Plant Ecol. 216, 307 (2015).

Laughlin, D. C., Fulé, P. Z., Huffman, D. W., Crouse, J. & Laliberté, E. Climatic constraints on trait-based forest assembly. J. Ecol. 99, 1489-1499 (2011).

Laughlin, D. C., Leppert, J. J., Moore, M. M. & Sieg, C. H. A multi-trait test of the leaf-height-seed plant strategy scheme with 133 species from a pine forest flora. Funct. Ecol. 24, 493-501 (2010).

Lavergne, S. & Molofsky, J. Increased genetic variation and evolutionary potential drive the success of an invasive grass. Proc. Natl Acad. Sci. U.S.A. 104, 3883-3888 (2007).

Lenters, T. P., Henderson, A., Dracxler, C. M., Elias, G. A., Kamga, S. M. et al. Integration and harmonization of trait data from plant individuals across heterogeneous sources. Ecol. Inform. 62, 101206 (2021).

Lhotsky, B., Csecserits, A., Kovács, B. & Botta-Dukát, Z. New plant trait records of the Hungarian flora. Li, R. et al. Are functional traits a good predictor of global change impacts on tree species abundance dynamics in a subtropical forest? Ecol. Lett. 18, 1181-1189 (2015).

Li, Y. & Shipley, B. Community divergence and convergence along experimental gradients of stress and disturbance. Ecology 99, 775-781 (2018).

Lin, D. et al. A plant economics spectrum of litter decomposition among coexisting fern species in a sub-tropical forest. Ann. Bot. 125, 145-155 (2020).

Lin, Y.-S., Medlyn, B. E., Duursma, R. A., Prentice, I. C., Wang, H. et al. Optimal stomatal behaviour around the world. Nat. Clim. Chang. 5, 459-464 (2015).

Losapio, G., De la Cruz, M., Escudero, A., Schmid, B. & Schöb, C. The assembly of a plant network in alpine vegetation. J. Veg. Sci. 29, 999-1006 (2018).

Louault, F., Pillar, V. D., Aufrere, J., Garnier, E. & Soussana, J. F. Plant traits and functional types in response to reduced disturbance in a semi-natural grassland. J. Veg. Sci. 16, 151-160 (2005).

Loveys, B. R., Atkinson, L. J., Sherlock, D. J., Roberts, R. L., Fitter, A. H. et al. Thermal acclimation of leaf and root respiration: an investigation comparing inherently fast- and slow-growing plant species. Glob. Change Biol. 9, 895-910 (2003).

Lukeš, P., Stenberg, P., Rautiainen, M., Mõttus, M. & Vanhatalo, K. M. Optical properties of leaves and needles for boreal tree species in Europe. Remote Sens. Lett. 4, 667-676 (2013).

Lusk, C. H. Leaf functional trait variation in a humid temperate forest, and relationships with juvenile tree light requirements. PeerJ 7, e6855 (2019).

Lusk, C. H., Kaneko, T., Grierson, E. & Clearwater, M. Correlates of tree species sorting along a temperature gradient in New Zealand rain forests: seedling functional traits, growth and shade tolerance. J. Ecol. 101, 1531-1541 (2013).

Maire, V., Wright, I. J., Prentice, I. C., Batjes, N. H., Bhaskar, R. et al. Global soil and climate effects on leaf photosynthetic traits and rates. Glob. Ecol. Biogeogr. 24, 706-717 (2015).

Manzoni, S., Vico, G., Porporato, A. & Katul, G. Biological constraints on water transport in the soil-plant-atmosphere system. Adv. Water Resour. 51, 292-304 (2013).

Markesteijn, L., Poorter, L., Paz, H., Sack, L. & Bongers, F. Ecological differentiation in xylem cavitation resistance is associated with stem and leaf structural traits. Plant Cell Environ. 34, 137-148 (2011).

Martin, A. R., Hale, C. E., Cerabolini, B. E. L., Cornelissen, J. H. C., Craine et al. Inter- and intraspecific variation in leaf economics traits in wheat and maize. AoB PLANTS, ply006 (2018).

Martin, R. E., Asner, G. P. & Sack, L. Genetic variation in leaf pigment, optical and photosynthetic function among diverse phenotypes of Metrosideros polymorpha grown in a common garden. Oecologia 151, 387-400 (2007).

Martin, S. M., Bonet, J. A., Martínez De Aragón, J., Voltas, J., Coll, L. et al. Crown bulk density and fuel moisture dynamics in Pinus pinaster stands are neither modified by thinning nor captured by the Forest Fire Weather Index. Ann. For. Sci. 74, 51 (2017).

Martinez-Garza, C., Bongers, F. & Poorter, L. Are functional traits good predictors of species performance in restoration plantings in tropical abandoned pastures? For. Ecol. Manage. 303, 35-45 (2013).

McCarthy, J. K., Mokany, K. & Dwyer, J. M. A regional-scale assessment of using metabolic scaling theory to predict ecosystem properties. Proc. R. Soc. B 286, 20192221 (2019).

McPartland, M. Alaska Peatland Experiment (APEX) PFT values (2016).

Medeiros, J. S., Burns, J. H., Nicholson, J., Rogers, L. & Valverde-Barrantes, O. Decoupled leaf and root carbon economics is a key component in the ecological diversity and evolutionary divergence of deciduous and evergreen lineages of genus Rhododendron. Am. J. Bot. 104, 803-816 (2017).

Medlyn, B. E., Badeck, F.-W., De Pury, D. G. G., Barton, C. V. M., Broadmeadow, M. et al. Effects of elevated CO2 on photosynthesis in European forest species: a meta-analysis of model parameters. Plant Cell Environ. 22, 1475-1495 (1999).

Meir, P. & Levy, P. E. Photosynthetic parameters from two contrasting woody vegetation types in West Africa. Plant Ecol. 192, 277-287 (2007).

Meir, P., Kruijt, B., Broadmeadow, M., Kull, O., Carswell, F. et al. Acclimation of photosynthetic capacity to irradiance in tree canopies in relation to leaf nitrogen concentration and leaf mass per unit area. Plant Cell Environ. 25, 343-357 (2002).

Mencuccini, M. The ecological significance of long distance water transport: short-term regulation and long-term acclimation across plant growth forms. Plant Cell Environ. 26, 163-182 (2003).

Méndez-Toribio, M., Ibarra-Manríquez, G., Paz, H. & Lebrija-Trejos, H. Atmospheric and soil drought risks combined shape tree species assembly in a Tropical Dry Forest. J. Ecol. (in press).

Messier, J., McGill, B. J. & Lechowicz, M. J. How do traits vary across ecological scales? A case for trait-based ecology. Ecol. Lett. 13, 838-848 (2010).

Michaletz, S. T. & Johnson, E. A. A heat transfer model of crown scorch in forest fires. Can. J. For. Res. 36, 2839-2851 (2006).

Michaletz, S. T., Weiser, M. D., McDowell, N. G., Zhou, J., Kaspari, M. et al. The energetic and carbon economic origins of leaf thermoregulation. Nat. Plants 2, 16129 (2016).

Michelaki, C., Fyllas, N. M., Galanidis, A., Aloupi, M., Evangelou, E. et al. An integrated phenotypic trait-network in thermo-Mediterranean vegetation describing alternative, coexisting resource-use strategies. Sci. Total Environ. 672, 583-592 (2019).

Milla, R. & Reich, P. B. Ann. Bot. 107, 455-465 (2011).

Miller, J. E. D., Ives, A. R., Harrison, S. P. & Damschen, E. I. Early- and late-flowering guilds respond differently to landscape spatial structure. J. Ecol. 106, 1033-1045 (2018).

Minden, V. & Kleyer, M. Testing the effect–response framework: key response and effect traits determining above-ground biomass of salt marshes. J. Veg. Sci. 22, 387-401 (2011).

Minden, V. & olde Venterink, H. Plant traits and species interactions along gradients of N, P and K availabilities. Funct. Ecol., accepted manuscript (2019).

Minden, V., Andratschke, S., Spalke, J., Timmermann, H. & Kleyer, M. Plant trait–environment relationships in salt marshes: deviations from predictions by ecological concepts. Perspect. Plant Ecol. Evol. Syst. 14, 183-192 (2012).

Minden, V., Deloy, A., Volkert, A. M., Leonhardt, S. D. & Pufal, G. Antibiotics impact plant traits, even at small concentrations. Ann. Bot. Plants 9, 1-19 (2017).

Minden, V., Schaller, J. & olde Venterink, H. Plants increase silicon content as a response to nitrogen or phosphorus limitation: a case study with Holcus lanatus. Plant Soil. (2020).

Moles, A. T., Falster, D. S., Leishman, M. R. & Westoby, M. Small-seeded species produce more seeds per square metre of canopy per year, but not per individual per lifetime. J. Ecol. 92, 384-396 (2004).

Mori, A. S. et al. Functional redundancy of multiple forest taxa along an elevational gradient: predicting the consequences of non-random species loss. J. Biogeogr. 42, 1383-1396 (2015).

Muller, S. C., Overbeck, G. E., Pfadenhauer, J. & Pillar, V. D. Plant functional types of woody species related to fire disturbance in forest-grassland ecotones. Plant Ecol. 189, 1-14 (2007).

Niinemets, Ü. & Valladares, F. Tolerance to shade, drought, and waterlogging of temperate Northern Hemisphere trees and shrubs. Ecol. Monogr. 76, 521-547 (2006).

Niinemets, Ü. Global-scale climatic controls of leaf dry mass per area, density, and thickness in trees and shrubs. Ecology 82, 453-469 (2001).

Nolan, R. H. et al. Divergence in plant water-use strategies in semiarid woody species. Funct. Plant Biol. 44, 1134-1146 (2017).

Núñez-Florez, R., Pérez-Gómez, U. & Fernández-Méndez, F. Functional diversity criteria for selecting urban trees. Urban For. Urban Green. 38, 251-266 (2019).

O'Reilly-Nugent, A., Wandrag, E. M., Catford, J. A., Gruber, B., Driscoll, D. et al. Measuring competitive impact: joint-species modelling of invaded plant communities. J. Ecol. 00, 1-11 (2019).

Ogaya, R. & Penuelas, J. Comparative field study of Quercus ilex and Phillyrea latifolia: photosynthetic response to experimental drought conditions. Environ. Exp. Bot. 50, 137-148 (2003).

Olson, M. E., Rosell, J. A., Martínez-Pérez, C., León-Gómez, C., Fajardo, A. et al. Xylem vessel diameter-shoot length scaling: ecological significance of porosity types and other traits. Ecol. Monogr. (in press).

Onoda, Y. et al. Global patterns of leaf mechanical properties. Ecol. Lett. 14, 301-312 (2011).

Onoda, Y. et al. Physiological and structural tradeoffs underlying the leaf economics spectrum. New Phytol. 214, 1447-1463 (2017).

Onstein, R. E., Carter, R. J., Xing, Y. et al. Diversification rate shifts in the Cape Floristic Region: The right traits in the right place at the right time. Perspect. Plant Ecol. Evol. Syst. 16, 331-340 (2014).

Onstein, R. E., Jordan, G. J., Sauquet, H., Weston, P. H., Bouchenak-Khelladi, Y. et al. Evolutionary radiations of Proteaceae are triggered by the interaction between traits and climates in open habitats. Glob. Ecol. Biogeogr. 25, 1239-1251 (2016).

Onstein, R. E., Kissling, W. D., Chatrou, L. W., Couvreur, T. L. P., Morlon, H. et al. Which frugivory-related traits facilitated historical long-distance dispersal in the custard apple family (Annonaceae)? J. Biogeogr. 46, 1874-1888 (2019).

Ordóñez, J. C., van Bodegom, P. M., Witte, J. P. M., Bartholomeus, R. P., van Hal, J. R. et al. Plant strategies in relation to resource supply in mesic to wet environments: does theory mirror nature? Am. Nat. 175, 225-239 (2010).

Paine, C. E. T. et al. Globally, functional traits are weak predictors of juvenile tree growth, and we do not know why. J. Ecol. 103, 978-989 (2015).

Pakeman, R. J., Brooker, R. W., Karley, A. J., Newton, A. C., Mitchell, C. et al. Increased crop diversity reduces the functional space available for weeds. Weed Res. 60, 121-131 (2020).

Pan, Y., Cieraad, E. & van Bodegom, P. M. Are ecophysiological adaptive traits decoupled from leaf economics traits in wetlands? Funct. Ecol. 33, 1202-1210 (2019).

Peco, B., de Pablos, I., Traba, J. & Levassor, C. The effect of grazing abandonment on species composition and functional traits: the case of dehesa. Basic Appl. Ecol. 6, 175-183 (2005).

Penuelas, J., Sardans, J., Llusia, J., Owen, S., Carnicer, J. et al. Faster returns on "leaf economics" and different biogeochemical niche in invasive compared with native plant species. Glob. Change Biol. 16, 2171-2185 (2010).

Perea, A. J., Garrido, J. L. & Alcántara, J. M. Plant functional traits involved in the assembly of canopy–recruit interactions. J. Veg. Sci. 32, e12991 (2021).

Perez, T. M., Rodriguez, J. & Heberling, J. M. Herbarium-based measurements reliably estimate three functional traits. Am. J. Bot. 107, 1457-1464 (2020).

Petter, G., Wagner, K., Zotz, G., Cabral, J. S., Wanek, W. et al. Distribution of functional leaf traits of vascular epiphytes: vertical trends, intra- and interspecific trait variability, and phylogenetic signals. Funct. Ecol. 30, 188–198 (2016).

Pierce, S., Brusa, G., Sartori, M. & Cerabolini, B. E. L. Combined use of leaf size and economics traits allows direct comparison of hydrophyte and terrestrial herbaceous adaptive strategies. Ann. Bot. 109, 1047-1053 (2012).

Pierce, S., Brusa, G., Vagge, I. & Cerabolini, B. E. L. Allocating CSR plant functional types: the use of leaf economics and size traits to classify woody and herbaceous vascular plants. Funct. Ecol. 27, 1002-1010 (2013).

Pierce, S., Ceriani, R. M., De Andreis, R., Luzzaro, A. & Cerabolini, B. The leaf economics spectrum of Poaceae reflects variation in survival strategies. Plant Biosyst. 141, 337-343 (2007).

Pierce, S., Luzzaro, A., Caccianiga, M., Ceriani, R. M. & Cerabolini, B. Disturbance is the principal α-scale filter determining niche differentiation, coexistence and biodiversity in an alpine community. J. Ecol. 95, 698-706 (2007).

Pierce, S., Vagge, I., Brusa, G. & Cerabolini, B. E. L. The intimacy between sexual traits and Grime's CSR strategies for orchids coexisting in semi-natural calcareous grassland at the Olive Lawn. Plant Ecol. 215, 495-505 (2014).

Pillar, V. D. & Sosinski, E. E. An improved method for searching plant functional types by numerical analysis. J. Veg. Sci. 14, 323-332 (2003).

Pomogaybin, A. V. & Pomogaybin, Ye. A. K izucheniyu bioekologicheskikh osobennostey predstaviteley roda Juglans L. pri introduktsii v lesostepi Srednego Povolzhya. Sovremennaya Botanika v Rossii, Trudy XIII syezda Russkogo Botanicheskogo Obshchestva, 156-158 (2014).

Poorter, H., Niinemets, Ü., Poorter, L., Wright, I. J. & Villar, R. Causes and consequences of variation in leaf mass per area (LMA): a meta-analysis. New Phytol. 182, 565-588 (2009).

Poorter, L. & Bongers, F. Leaf traits are good predictors of plant performance across 53 rain forest species. Ecology 87, 1733-1743 (2006).

Poorter, L. Leaf traits show different relationships with shade tolerance in moist versus dry tropical forests. New Phytol. 181, 890-900 (2009).

Poschlod, P., Kleyer, M., Jackel, A. K., Dannemann, A. & Tackenberg, O. BIOPOP - a database of plant traits and Internet application for nature conservation. Folia Geobot. 38, 263-271 (2003).

Powers, J. S. & Tiffin, P. Plant functional type classifications in tropical dry forests in Costa Rica: leaf habit versus taxonomic approaches. Funct. Ecol. 24, 927-936 (2010).

Prentice, I. C., Meng, T., Wang, H., Harrison, S. P. et al. Evidence for a universal scaling relationship of leaf CO2 drawdown along a moisture gradient. New Phytol. 190, 169-180 (2011).

Preston, K. A., Cornwell, W. K. & DeNoyer, J. L. Wood density and vessel traits as distinct correlates of ecological strategy in 51 California coast range angiosperms. New Phytol. 170, 807-818 (2006).

Price, C. A. & Enquist, B. J. Scaling of mass and morphology in dicotyledonous leaves: an extension of the WBE model. Ecology 88, 1132-1141 (2007).

Purcell, A. S. T., Lee, W. G., Tanentzap, A. J. & Laughlin, D. C. Fine root traits are correlated with flooding duration while aboveground traits are related to grazing in an ephemeral wetland. Wetlands 39, 291-302 (2019).

Pyankov, V. I., Kondratchuk, A. V. & Shipley, B. Leaf structure and specific leaf mass: the alpine desert plants of the Eastern Pamirs, Tadjikistan. New Phytol. 143, 131-142 (1999).

Quero, J. L., Villar, R., Maranon, T., Zamora, R., Vega, D. et al. Relating leaf photosynthetic rate to whole-plant growth: drought and shade effects on seedlings of four Quercus species. Funct. Plant Biol. 35, 725-737 (2008).

Quested, H. M., Cornelissen, J. H. C., Press, M. C., Callaghan, T. V., Aerts, R. et al. Decomposition of sub-arctic plants with differing nitrogen economies: a functional role for hemiparasites. Ecology 84, 3209-3221 (2003).

Quitián, R. et al. Functional traits in Oecologia. Oecologia 189, 435-445 (2019).

Raevel, V., Anthelme, F., Meneses, R. I. & Munoz, F. Cushion-plant protection determines guild-dependent plant strategies in high-elevation peatlands of the Cordillera Real, Bolivian Andes. Perspect. Plant Ecol. Evol. Syst. 30, 103-114 (2018).

Raevel, V., Munoz, F., Pons, V., Renaux, A., Martin, A. et al. Changing assembly processes during a primary succession of plant communities on Mediterranean roadcuts. J. Plant Ecol. 6, 19-28 (2013).

Raevel, V., Violle, C. & Munoz, F. Mechanisms of ecological succession: insights from plant functional strategies. Oikos 121, 1761-1770 (2012).

Reich, P. B., Oleksyn, J. & Wright, I. J. Leaf phosphorus influences the photosynthesis-nitrogen relation: a cross-biome analysis of 314 species. Oecologia 160, 207-212 (2009).

Reich, P. B., Tjoelker, M. G., Pregitzer, K. S., Wright, I. J., Oleksyn, J. et al. Scaling of respiration to nitrogen in leaves, stems and roots of higher land plants. Ecol. Lett. 11, 793-801 (2008).

Reichenau, T. G., Korres, W., Schmidt, M., Graf, A., Welp, G. et al. A comprehensive dataset of vegetation states, fluxes of matter and energy, weather, agricultural management, and soil properties from intensively monitored crop sites in western Germany. Earth Syst. Sci. Data 12, 2333-2350 (2020).

Rodrigues, A. V., Bones, F. L. V., Schneiders, A., Oliveira, L. Z., Vibrans, A. C. et al. Plant trait dataset for tree-like growth forms species of the subtropical Atlantic Rain Forest in Brazil. Data 3, 16 (2018).

Rogers, A., Serbin, S. P., Ely, K. S., Sloan, V. L. & Wullschleger, S. D. Terrestrial biosphere models underestimate photosynthetic capacity and CO2 assimilation in the Arctic. New Phytol. (2017).

Rolo, V., Olivier, P. & van Aarde, R. Seeded pioneer die-offs reduce the functional trait space of new-growth coastal dune forests. For. Ecol. Manage. 377, 26-35 (2016).

Ronzhina, D. A. & Pyankov, V. I. Structure of the photosynthetic apparatus in leaves of freshwater hydrophytes: 1. General characteristics of the leaf mesophyll and a comparison with terrestrial plants. Russ. J. Plant Physiol. 48, 567–575 (2001).

Rossi, C., Kneubuehler, M., Schuetz, M., Shaepman, M. E., Haller, R. M. et al. From local to regional: functional diversity in differently managed alpine grasslands. Remote Sens. Environ. 236, 111415 (2020).

Sack, L. Responses of temperate woody seedlings to shade and drought: do trade-offs limit potential niche differentiation? Oikos 107, 110-127 (2004).

Sack, L., Cowan, P. D., Jaikumar, N. & Holbrook, N. M. The 'hydrology' of leaves: co-ordination of structure and function in temperate woody species. Plant Cell Environ. 26, 1343-1356 (2003).

Sack, L., Melcher, P. J., Liu, W. H., Middleton, E. & Pardee, T. How strong is intracanopy leaf plasticity in temperate deciduous trees? Am. J. Bot. 93, 829-839 (2006).

Sack, L., Tyree, M. T. & Holbrook, N. M. Leaf hydraulic architecture correlates with regeneration irradiance in tropical rainforest trees. New Phytol. 167, 403-413 (2005).

Sancho-Knapik, D., Escudero, A., Mediavilla, S., Scoffoni, C., Zaila, J. et al. Deciduous and evergreen oaks show contrasting adaptive responses in leaf mass per area across environments. (to be submitted).

Sandel, B., Corbin, J. D. & Krupa, M. Using plant functional traits to guide restoration: a case study in California coastal grassland. Ecosphere 2, art23 (2011).

Scalon, M. C. et al. Diversity of functional trade-offs enhances survival after fire in Neotropical savanna species. J. Veg. Sci. (in press).

Scalon, M. C., Haridasan, M. & Franco, A. C. Plant Soil (2017).

Schall, P., Lödige, C., Beck, M. & Ammer, C. Biomass allocation to roots and shoots is more sensitive to shade and drought in European beech than in Norway spruce seedlings. For. Ecol. Manage. 266, 246-253 (2012).

Scherer-Lorenzen, M., Schulze, E.-D., Don, A., Schumacher, J. & Weller, E. Exploring the functional significance of forest diversity: A new long-term experiment with temperate tree species (BIOTREE). Perspect. Plant Ecol. Evol. Syst. 9, 53-70 (2007).

Schmitt, S., Raevel, V., Rejou-Mechain, M., Ayyappan, N., Balachandran, N., Barathan, N. et al. Canopy and understorey tree guilds respond differently to the environment in an Indian rain forest. J. Veg. Sci. 32, 1-9 (2021).

Schrader, J., Moeljono, S., Tambing, J., Sattler, C., Kreft, H. et al. A new dataset on plant occurrences on small islands, including species abundances and functional traits across different spatial scales. Biodivers. Data J. 8, e55275 (2020).

Schroeder-Georgi, T., Wirth, C., Nadrowski, K., Meyer, S. T., Mommer, L. & Weigelt, A. From pots to plots: hierarchical trait-based prediction of plant performance in a mesic grassland. J. Ecol. 104, 206-218 (2016).

Schuldt, B., Leuschner, C., Brock, N. & Horna, V. Tree Physiol. 33, 161-174 (2013).

Schurr, F. M., Midgley, G. F., Rebelo, A. G., Reeves, G., Poschlod, P. et al. How to understand species' range shifts. Glob. Ecol. Biogeogr. 16, 449-459 (2007).

Schweingruber, F. H. & Landolt, W. The Xylem Database. Swiss Federal Research Institute WSL, updated (2005).

Scoffoni, C., Pou, A., Aasamaa, K. & Sack, L. The rapid light response of leaf hydraulic conductance: new evidence from two experimental methods. Plant Cell Environ. 31, 1803-1812 (2008).

Sharpe, J. M. & Solano, N. Traits of fertile (spore-bearing) leaves of understory rainforest ferns from the El Verde Field Station in El Yunque National Forest, Puerto Rico, USA. Unpublished data (2016).

Sharpe, J. M. & Solano, N. Traits of sterile (non-spore-bearing) leaves of understory rainforest ferns from the El Verde Field Station in El Yunque National Forest, Puerto Rico, USA, and Monteverde cloud forest, Costa Rica. Unpublished data (2016).

Shiodera, S., Rahajoe, J. S. & Kohyama, T. Variation in longevity and traits of leaves among co-occurring understorey plants in a tropical montane forest. J. Trop. Ecol. 24, 121-133 (2008).

Shipley, B. & Lechowicz, M. J. The functional co-ordination of leaf morphology, nitrogen concentration, and gas exchange in 40 wetland species. Ecoscience 7, 183-194 (2000).

Shipley, B. & Vu, T. T. Dry matter content as a measure of dry matter concentration in plants and their parts. New Phytol. 153, 359-364 (2002).

Shipley, B. Structured interspecific determinants of specific leaf area in 34 species of herbaceous angiosperms. Funct. Ecol. 9, 312-319 (1995).

Shipley, B. Trade-offs between net assimilation rate and specific leaf area in determining relative growth rate: relationship with daily irradiance. Funct. Ecol. 16, 682-689 (2002).

Siefert, A. Spatial patterns of functional divergence in old-field plant communities. Oikos 121, 907-914 (2012).

Siefert, A., Fridley, J. D. & Ritchie, M. E. Community functional responses to soil and climate at multiple spatial scales: when does intraspecific variation matter? PLoS ONE 9, e111189 (2014).

Silva, M. C., Teodoro, G. S., Bragion, E. F. A. & van den Berg, E. The role of intraspecific trait variation in the occupation of sharp forest-savanna ecotones. Flora 253, 35-42 (2019).

Slot, M., Rey-Sanchez, C., Winter, K. & Kitajima, K. Trait-based scaling of temperature-dependent foliar respiration in a species-rich tropical forest canopy. Funct. Ecol. 28, 1074-1086 (2014).

Smith, N. G. & Dukes, J. S. LCE: leaf carbon exchange data set for tropical, temperate, and boreal species of North and Central America. Ecology 98, 2978 (2017).

Smith, S. W., Woodin, S. J., Pakeman, R. J., Johnson, D. & van der Wal, R. Root traits predict decomposition across a landscape-scale grazing experiment. New Phytol. 202, 157-169 (2014).

Sodhi, D. S., Livingstone, S. W., Carboni, M. & Cadotte, M. W. Plant invasion alters trait composition and diversity across habitats. Ecol. Evol. 9, 6199-6210 (2019).

Soudzilovskaia, N. A. et al. Functional traits predict relationship between plant abundance dynamics and long-term climate warming. Proc. Natl Acad. Sci. U.S.A. 110, 18180-18185 (2013).

Soudzilovskaia, N. A., Elumeeva, T. G., Onipchenko, V. G., Shidakov, I. I., Salpagarova, F. S. et al. Functional traits predict relationship between plant abundance dynamics and long-term climate warming. Proc. Natl Acad. Sci. U.S.A. 110, 18180-18184 (2013).

Souza, J. P., Melo, N. M. J., Pereira, E. G., Halfeld, A. D., Gomes, I. N. et al. Responses of woody Cerrado species to rising atmospheric CO2 concentration and water stress: gains and losses. Funct. Plant Biol. 43, 1183-1193 (2016).

Souza, K., Higuchi, P., Silva, A. C., Schimalski, M. B., Loebens, R. et al. Partição de nicho por grupos funcionais de espécies arbóreas em uma floresta subtropical. Rodriguésia 68, 1165-1175 (2017).

Spasojevic, M. J. & Suding, K. N. Inferring community assembly mechanisms from functional diversity patterns: the importance of multiple assembly processes. J. Ecol. 100, 652-661 (2012).

Spasojevic, M. J., Turner, B. L. & Myers, J. A. When does intraspecific trait variation contribute to functional beta-diversity? J. Ecol. 104, 487-496 (2016).

Staples, T. L., Dwyer, J. M., England, J. R. & Mayfield, M. M. Productivity does not correlate with species and functional diversity in Australian reforestation plantings across a wide climate gradient. Glob. Ecol. Biogeogr. 28, 1417-1429 (2019).

Swaine, E. K. Ecological and evolutionary drivers of plant community assembly in a Bornean rain forest. PhD thesis, University of Aberdeen (2007).

Swenson, N. G., Anglada-Cordero, P. & Barone, J. A. Deterministic tropical tree community turnover: evidence from patterns of functional beta diversity along an elevational gradient. Proc. R. Soc. B 278, 877-884 (2011).

Takkis, K. Changes in plant species richness and population performance in response to habitat loss and fragmentation. Dissertationes Biologicae Universitatis Tartuensis 255 (2014).

Takkis, K., Saar, L., Pärtel, M. & Helm, A. Effect of environment and landscape on the traits of six plant species in fragmented grasslands. (in preparation)

Tang, X., Liu, D., Tu, B., Yang, X. & Yang, X. Promotion effect on growth of mycorrhiza-inoculated mulberry saplings and physiological and biochemical mechanism to drought tolerance. J. Southwest Univ. 35, 19-26 (2013).

Tavsanoglu, C. & Pausas, J. G. A functional trait database for Mediterranean Basin plants. Sci. Data 5, 180135 (2018).

Terziyska, T. S., Tsakalos, J. L., Bartha, S., Apostolova, I., Sopotlieva, D. et al. Species and functional differences between subalpine grasslands with and without dwarf shrub encroachment. Plant Biosyst. 1-10 (2019).

Thomas, E., Alcazar, C., Moscoso, L. G., Osorio, L. F., Salgado, B. et al. The importance of species selection and seed sourcing in forest restoration for enhancing adaptive potential to climate change: Colombian tropical dry forest as a model. CBD Tech. Ser. 89, 122-134 (2017).

Thuiller, W. Traits of European Alpine Flora. OriginAlps Project, CNRS.

Tng, D. Y. P., Jordan, G. J. & Bowman, D. M. J. S. Plant traits demonstrate that temperate and tropical giant eucalypt forests are ecologically convergent with rainforest, not savanna. PLoS ONE 8, e84378 (2013).

Toledo-Aceves, T., García-Hernández, M. & Paz, H. Leaf functional traits predict cloud forest tree seedling survival along an elevation gradient. Ann. For. Sci. 76, 111 (2019).

Tribouillois, H., Fort, F., Cruz, P., Charles, R., Flores, O. et al. A functional characterisation of a wide range of cover crop species: growth and nitrogen acquisition rates, leaf traits and ecological strategies. PLoS ONE 10, e0122156 (2015).

Tucker, S. S., Craine, J. M. & Nippert, J. B. Physiological drought tolerance and the structuring of tallgrass assemblages. Ecosphere 2, 48 (2011).

Utaile, Y. U., Honnay, O., Muys, B., Cheche, S. S. & Helsen, K. Effect of Dichrostachys cinerea encroachment on plant species diversity, functional traits and litter decomposition in an East-African savannah ecosystem. J. Veg. Sci. 00, 1–12 (2020).

van Bodegom, P. M., Sorrell, B. K., Oosthoek, A., Bakke, C. & Aerts, R. Separating the effects of partial submergence and soil oxygen demand on plant physiology. Ecology 89, 193-204 (2008).

Van Cleemput, E., Roberts, D., Honnay, O. & Somers, B. A novel procedure for measuring functional traits of herbaceous species through field spectroscopy. Methods Ecol. Evol. (2019).

van de Weg, M. J., Meir, P., Grace, J. & Atkin, O. Altitudinal variation in leaf mass per unit area, leaf tissue density and foliar nitrogen and phosphorus content along the Amazon-Andes gradient in Peru. Plant Ecol. Divers. 2, 243-254 (2009).

van de Weg, M. J., Meir, P., Grace, J. & Ramos, G. D. Photosynthetic parameters, dark respiration and leaf traits in the canopy of a Peruvian tropical montane cloud forest. Oecologia. (2011).

van der Merwe, S., Greve, M., Olivier, B. & le Roux, P. C. The role of facilitation in functional trait expression: testing the generality in two contrasting systems. (in prep).

van der Plas, F. & Olff, H. Mesoherbivores affect grasshopper communities in a megaherbivore-dominated South African savannah. Oecologia 175, 639-649 (2014).

van der Sande, M. T., Arets, E. J. M. M., Peña-Claros, M., Hoosbeek, M. R., Cáceres-Siani, Y. et al. Soil fertility and species traits, but not diversity, drive productivity and biomass stocks in a Guyanese tropical rainforest. Funct. Ecol. 32, 461-474 (2018).

Vanselow, K. A., Samimi, C. & Breckle, S.-W. Preserving a comprehensive vegetation knowledge base: an evaluation of four historical Soviet vegetation maps of the Western Pamirs. PLoS ONE 11, e0148930 (2016).

Vásquez-Valderrama, M. Efecto de especies con potencial invasor en procesos de regulación hídrica del suelo en un ecosistema seco tropical. Universidad Distrital Francisco José de Caldas, Maestría en Manejo, Uso y Conservación del Bosque, Bogotá, Colombia (2016).

Verdier, B., Jouanneau, I., Simonnet, B., Rabin, C., Van Dooren, T. J. et al. Climate and atmosphere simulator for experiments on ecological systems in changing environments. Environ. Sci. Technol. 48, 8744-8753 (2014).

Vergutz, L., Manzoni, S., Porporato, A., Novais, R. F. & Jackson, R. B. A global database of carbon and nutrient concentrations of green and senesced leaves. ORNL DAAC (2012).

Vile, D. Significations fonctionnelle et ecologique des traits des especes vegetales: exemple dans une succession post-cultural mediterraneenne et generalisations. PhD Thesis (2005).

Von Holle, B. & Simberloff, D. Testing Fox's assembly rule: Does plant invasion depend on recipient community structure? Oikos 105, 551-563 (2004).

Wagenführ, R. Holzatlas. 6., neu bearbeitete und erweiterte Auflage (Fachbuchverlag Leipzig, 2007).

Walker, A. P. A Global Data Set of Leaf Photosynthetic Rates, Leaf N and P, and Specific Leaf Area. Oak Ridge National Laboratory Distributed Active Archive Center, Oak Ridge, TN, USA (2014).

Wang, H., Harrison, S. P., Prentice, I. C., Yang, Y., Bai, F. et al. The China Plant Trait Database. PANGAEA (2017).

Wei, L. & Fenton, N. J. Stem specific density and specific leaf area measured for project "Silviculture based on plant functional traits in boreal forests". Université de Québec en Abitibi-Témiscamingue, Unpublished data (2016).

Wellstein, C., Chelli, S., Campetella, G., Bartha, S., Galiè, M. et al. Intraspecific phenotypic variability of plant functional traits in contrasting mountain grasslands habitats. Biodivers. Conserv. 22, 2353-2374 (2013).

White, M. A., Thornton, P. E., Running, S. W. & Nemani, R. R. Parameterization and sensitivity analysis of the BIOME-BGC terrestrial ecosystem model: Net primary production controls. Earth Interactions 4, 1-85 (2000).

Williams, M., Shimabokuro, Y. E. & Rastetter, E. B. LBA-ECO CD-09 Soil and Vegetation Characteristics, Tapajos National Forest, Brazil. Oak Ridge Natl. Lab. DAAC (2012).

Willis, C. G., Halina, M., Lehman, C., Reich, P. B., Keen, A. et al. Phylogenetic community structure in Minnesota oak savanna is influenced by spatial extent and environmental variation. Ecography 33, 565-577 (2010).

Wilson, K., Baldocchi, D. & Hanson, P. Spatial and seasonal variability of photosynthetic parameters and their relationship to leaf nitrogen in a deciduous forest. Tree Physiol. 20, 565–578 (2000).

Wirth, C. & Lichstein, J. W. The imprint of species turnover on old-growth forest carbon balances - insights from a trait-based model of forest dynamics. In Wirth, C., Gleixner, G. & Heimann, M. (eds.) Old-Growth Forests: Function, Fate and Value, 81-113 (Springer, New York, Berlin, Heidelberg, 2009).

Wright, I. J. et al. Global climatic drivers of leaf size. Science 357, 917-921 (2017).

Wright, I. J., Ackerly, D. D., Bongers, F., Harms, K. E., Ibarra-Manriquez, G. et al. Relationships among ecologically important dimensions of plant trait variation in seven Neotropical forests. Ann. Bot. 99, 1003-1015 (2007).

Wright, I. J., Cooke, J., Cernusak, L. A., Hutley, L. B., Scalon, M. C. et al. Stem diameter growth rates in a fire-prone savanna correlate with photosynthetic rate and branch-scale biomass allocation, but not specific leaf area. Austral Ecol. 44, 339-350 (2019).

Wright, I. J., Reich, P. B., Westoby, M., Ackerly, D. D., Baruch, Z. et al. The worldwide leaf economics spectrum. Nature 428, 821-827 (2004).

Wright, J. P. & Sutton-Grier, A. Does the leaf economic spectrum hold within local species pools across varying environmental conditions? Funct. Ecol. (2012).

Wright, S. J., Kitajima, K., Kraft, N. J. B., Reich, P. B., Wright, I. J. et al. Functional traits and the growth-mortality tradeoff in tropical trees. Ecology 91, 3664-3674 (2011).

Xin-e, L., Nie, Y., Song, X., Zhang, R. & Wang, G. Patterns of species diversity and functional diversity along a south-to north-facing slope in a sub-alpine meadow. Community Ecol. 12, 179-187 (2011).

Zanne, A. E. et al. Three keys to the radiation of angiosperms into freezing environments. Nature 506, 89-92 (2013).

Zheng, J., Zang, H., Yin, S., Sun, N., Zhu, P. et al. Modeling height-diameter relationship for artificial monoculture Metasequoia glyptostroboides in sub-tropic coastal megacity Shanghai, China. Urban For. Urban Green. 34, 226-232 (2018).

Zheng, W. Silva Sinica: Volume 1-4 (China Forestry Publishing House, Beijing, 1983).

Ziemińska, K., Butler, D. W., Gleason, S. M., Wright, I. J. & Westoby, M. Fibre wall and lumen fractions drive wood density variation across 24 Australian angiosperms. AoB Plants 5 (2013).

Zirbel, C. R., Bassett, T., Grman, E. & Brudvig, L. A. Data from: plant functional traits and environmental conditions shape community assembly and ecosystem functioning during restoration. Dryad Digital Repository (2017).

Zirbel, C. R., Bassett, T., Grman, E. & Brudvig, L. A. Plant functional traits and environmental conditions shape community assembly and ecosystem functioning during restoration. J. Appl. Ecol. 54, 1070-1079 (2017).
